# Are small farms more performant than larger ones in developing countries?

**DOI:** 10.1126/sciadv.abb8235

**Published:** 2020-10-09

**Authors:** P. A. Garzón Delvaux, L. Riesgo, S. Gomez y Paloma

**Affiliations:** 1 European Commission, Joint Research Centre (JRC), 41092 Seville, Spain.; 2 Universidad Pablo de Olavide, Department of Economics, 41013 Seville, Spain.

## Abstract

Meta-regressions of around 1000 cases published over the period 1997–2018 suggest that the direction of the relationship between land area and agricultural performance strongly depends on the performance indicator selected. Net value and efficiency indicators show that larger farms tend to be more performant than smallholders, while the simpler but ubiquitous gross output indicators support an inverse relationship (IR). In addition, this study also indicates a decreasing record of IR in the literature over time, regardless of the indicator used. This may be partially explained by improvements in assessment techniques but, more importantly, by agricultural structural changes. Our results invite reconsidering IR as a central assumption when formulating agricultural support in rural development policy.

## Teaser

Small farms tend to be less profitable and efficient than larger ones, questioning development strategies in the long run.

## INTRODUCTION

Smallholder farming is crucial for producing food and sustaining millions of livelihoods in developing countries. In low-income and lower middle–income countries, farms managing less than 2 ha make up about 84% of all farms and operate about 30 to 40% of available land (*1*); the labor force is critically dependent on them (*2*, *3*). Smallholders are also highly substantial to one of the main staple crops worldwide by producing over 65% of all rice (*4*), along with several agricultural export commodities such as cocoa, coffee, tea, rubber, and palm oil (*5*, *6*).

The relationship between land size (i.e., farm size or, in some cases, plot size) and output per area has been widely discussed in the literature, contrasting trends in developing and developed countries (*7*–*9*). The rising average size of farm holdings in the developed world (*1*, *8*) has been associated with both large improvements in crop productivity (output per unit of land) (*10*) and overall level of development (*11*) over the past century. By contrast, an inverse relationship (IR) between land area and output per unit of land (productivity) has been highlighted as a recurrent phenomenon in developing economies, where, in most of the cases, the average farm size has been declining over the same period (*1*, *8*, *12*). Since its initial observation in early Soviet agriculture (*13*), smaller farms have been consistently recorded as producing more per area than larger holdings, first in South Asia throughout the 1960s and 1970s (*14*–*19*) and afterward in the whole region as the Asian Green Revolution unfolded (*8*, *12*). Examples from Latin America were also recorded such as for Brazil (*20*, *21*). More recently, a similar trend has been reported in Sub-Saharan Africa (SSA) (*22*, *23*), probably due to the more recent intensification of agriculture (*24*).

The main justifications for this higher incidence of IR in developing contexts are related to the presence of imperfections in labor, inputs, financial, and/or land markets (*14*, *25*–*28*). As first introduced by Sen (*15*) in the post-Second World War literature, a strong candidate for the contextual explanation of a systematic IR in development context is that of labor market imperfections hypothesis. A fundamental mechanism is that when agricultural wage is sufficiently low (or almost null in given family labor circumstances), the family labor–intensive agriculture is more competitive (*13*, *14*, *17*, *29*). In an alternative explanation, other authors (*16*, *26*, *30*, *31*) justify IR through labor supervision costs, making hired labor expensive relative to family labor when land market is imperfect. Hence, IR is observed among more efficient small-scale farms and inefficient large farms coexisting. Although imperfections in only one factor market may not be sufficient to introduce a systematic relationship between farm size and performance, if two or more coalesce, then such a relationship is expected, particularly IR (*9*). For example, even if labor markets are imperfect but land markets do allow transfer of (at least) long-term user rights from land-abundant households to labor-abundant ones, the relationship will not show IR. When one of the markets is competitive (and the production function is subject to constant returns to scale), an equally efficient allocation of resources among farms can be achieved in equilibrium. If labor markets work, then labor-abundant but land-scarce households will work for land-abundant households. In turn, if land markets operate (either as land sales or rent), then labor-abundant households will either purchase or rent land from land-abundant ones (*24*). Other authors (*25*) show that imperfections in land markets and the absence of insurance markets are responsible of oversupplying labor on their small farms in an attempt of small farmers (usually net buyers of staple crops) to reduce the risk of price fluctuations when buying from a market. However, in most developing countries, neither labor land nor insurance markets function efficiently, favoring the apparition of IR and limiting the performance of larger operation in relation to smallholders.

This evidence of IR has been translated into supporting the smallholder strategies of international organizations and aid donors in response to development challenges *(**2*, *32*–*34**)*. If small farms truly outperform larger ones, then there would be no clear trade-off between equity and efficiency in the design and implementation of development policies: Supporting poorer farmers would also enhance aggregate productivity (*35*). However, recent literature points to weakening of this evidence in some developing countries (*28*, *36*–*39*) or simply records that as the size of farms increases, so does the productivity (*40*, *41*). As a result, the direction of the farm size–performance relationship in developing countries cannot be inherently assumed (*8*, *42*, *43*). Several explanations have been proposed to shed light on the potential overdetection of IR. One of the possible reasons for the prevalence of IR in the literature has been methodological weaknesses related to misspecification of econometric models, through failing to account for fixed effects such as the quality of soils or community characteristics (*22*, *44*–*48*), or lack of accurate measurement of production and/or land size due to relying only on farmers’ perceptions (*48*–*51*) or to inappropriateness of the productivity measure used in the debate (*9*, *35*, *43*, *48*).

As pointed out recently (*52*), the range of land size surveyed may also influence assessment of the relationship between land size and agricultural performance. In nondensely populated countries, studies based on survey data [e.g., analyses using Living Standards Measurement Study–Integrated Surveys on Agriculture (LSMS-ISA)] focus mainly on farms under 10 ha, which may result in biased estimates of the relationship and its nature (*53*, *54*).

Acknowledging the limitations in assessing the relationship between agricultural land size and productivity in developing countries, we aim to shed light on the existing evidence, as well as the salient drivers of this relationship. Our meta-analysis complements earlier reviewing efforts from a more qualitative perspective (*35*, *55*). Both articles discuss the state of the art about the reasons explaining IR, with (*35*) also discussing the different indicators used in the literature to measure size of holdings and productivity. In this sense, Gollin (*35*) also shows some recent evidence on farm size and productivity among different developing countries, focusing the discussion on labor productivity differences. In addition, the study of Larson *et al.* (*55*) relates the potential of small farms to be engaged on speeding economic growth through their productivity improvement, as well as their relationship with food security and poverty alleviation, comparing Asian and SSA realities.

More specifically, our quantitative meta-study builds on the existing studies of the agricultural performance–land size relationship in at least the following aspects. First, it provides the probability of finding different land size–performance relationships in the literature (at farm and plot levels), the possibility of comparing this likelihood by indicator, and how a number of variables may influence on the result (variables related to model specification, main crop, or bibliometric issues). Second, it offers a long view as to the evolution in time of IR prevalence: It is diminishing over the 22-year period reviewed in the literature, even when considering the type of indicator used. This confirms isolated trends identified by single case studies lacking global perspective. Last, it widens the literature traditionally referred to in the debate (i.e., specialized literature) and integrates results from relevant but neglected evidence (e.g., Ricardian analyses for climate change impact). The selection process also includes selected econometric exercises, which did not specifically intent to analyze the relationship between farm size and agricultural performance but which control for key parameters such as quality of soil or market imperfections along farm/plot area to analyze farm performance indicators. This allows controlling for potential publication bias in the specialized literature.

We collected empirical evidence documenting the relationship between agricultural land size and productivity in the developing world, published in peer-reviewed journals and reports from relevant institutions [World Bank, International Food Policy Research Institute (IFPRI), etc.] between 1997 and 2018. The resulting unique dataset includes information both from case studies focusing on the land size–performance relationship (hereafter referred as “specialized literature”) and from others covering general productivity estimations that also accounted for land size (referred to as “nonspecialized” or “generalist” literature).

This study reports findings from a meta-regression with over 1000 cases in the developing world including the different land management scales that can be identified in the literature: farm and plot. In addition, two different approaches are analyzed to compare results: (i) a nonweighted analysis of all the cases (i.e., all the cases collected have the same weight in the analysis) and (ii) a weighted analysis reflecting the quality of the publication (i.e., more weight is given to information provided by studies published in top ranking peer-reviewed scientific journals).

We demonstrate that the choice of indicator used for measuring productivity has clear consequences for the probability of detecting IR. Analyses using economic indicators related to efficiency, or total factor productivity, systematically report less IR than gross output indicators frequently used in the literature (e.g., yields), at both farm and plot levels. The probability of recording IR when using net value and efficiency indicators is reduced by at least 24% compared to the probability when using gross output indicators, implying that IR is not as widespread as currently assumed in the policy debate.

Looking at the subsample of papers analyzing at least two different indicators for the same country and region, crop and unit of analysis (farm/plot), results show that within a paper where gross output and net value indicators can be compared, 60% of cases show the same result (IR prevalence) but 40% of the cases show different results between indicators (IR prevalence using gross output and direct relationship using net value). Therefore, results do not show a clear difference when using gross output or net value indicators in the subsample of papers, whereas on the whole subsample, this difference exists and is statistically significant but weak.

However, when comparing results from gross output and efficiency indicators within a paper, we can see that differences prevail in 69% of cases (gross output points to an IR, whereas efficiency indicates to a direct relationship). In this case, the subsample points to the same trend as the whole dataset of papers, and a clear difference appears when using gross output and efficiency indicators. This pattern is explained mainly by the intensity of family labor use [as reported by (*11*) for 55 countries as farms use mainly household member workers and very limited hired labor]. This results in IR when looking at gross output indicators, but this intensive use of labor makes the advantage on gross output disappear when using efficiency indicators (the use of the labor factor is higher in small than in larger farms). Although expected from a theoretical point of view and confirmed in industrialized contexts, the literature exploring how labor use influences efficiency of small/larger farms is less abundant for developing countries. Hence, this meta-analysis contributes to recognition of this gap and to advocacy for a more informed approach by development practitioners.

Another key result is that the likelihood of recording IR across all indicators decreases with time. Results also show the relevance of the data source for the studies (e.g., LSMS-ISA, national statistics, or ad hoc surveys) to the likelihood of detecting IR. Other variables tested show some influence on the probability of recording IR, when scaling the analysis at plot level. Studies at plot level from the specialized literature seem more likely to record IR than generalist studies. Last, plot-level studies controlling for soil quality tend to be less likely to record IR than those that do not. This is also identified when exclusively looking at the subsample of farm studies using gross output as a performance indicator.

## MATERIALS AND METHODS

### Scope, database construction, and unit of analysis

The scope of the analysis covers the developing world dividing into microregion [i.e., SSA, Middle East and North Africa (MENA), Asia, and Latin America and the Caribbean (LAC)] and most of its agroecological zones, as of available information. The review only considered econometric exercises, which assessed agricultural land performance (through various indicators), at least by including land size as an explanatory variable. Simple descriptive studies were not included, along other exclusion. Searches were conducted with the use of keywords, followed by filtering using exclusion and inclusion criteria. Details of the process are provided in the Supplementary Materials (see sections S2 to S4).

The sample of studies providing land size–performance data was gathered through an extensive analysis of peer-reviewed publications, in English and French, over 22 years (1997–2018). Material identified in Spanish, Portuguese, and Italian was also explored. Searches were conducted in Scopus and the Cairn repository for additional French publications. In addition, the institutional repositories of the World Bank, FAO (Food and Agriculture Organization of the United Nations), IFPRI, and CIRAD (Centre de coopération internationale en recherche agronomique pour le développement) were explored. The material was completed through snowballing, adding references identified in relevant papers.

Searches and coding were based on PRISMA/PRISMA-P (Preferred Reporting Items for Systematic Review and Meta-Analysis Protocols) (*56*, *57*) and MAER-NET (Meta-Analysis of Economics Research Network) guidelines (*58*). Hence, data coding was performed by the first two authors, using revisions and consultations throughout the process to refine the database. This process was particularly relevant, given the methodological and contextual variety of studies, spanning the globe and over two decades.

We identified 474 relevant papers, yielding a pool of a total of 1135 distinct cases, at farm and plot levels (see section S1 for the identification method of cases in each paper and section S5 for full reference on the material gathered). The period selected provides the base for a consistent approach to research material (surveying/econometric techniques), with a broad geographical coverage and long enough to capture variations through time.

### Variables

The compiled database contains entries detailing bibliographic information, our dependent variable (i.e., land size–agricultural performance relationship), indicators to account for land performance, average land area in the study, and data source. It also details key variables explaining performance such as main crops, agroecological zone, and characteristics of the model specification related to the inclusion of control variables [e.g., soil quality, Global Positioning System (GPS) measurement, access to irrigation, access to credit, or use of animal or mechanical power]. Although family labor was considered a priori as a relevant variable for the study, the difficulties to identify the actual scope of the labor variable (e.g., family, hired, a mixed of both) included in the original analyses, prevented us to use it. Moreover, the fact that it is ubiquitous makes it less informative as meta-data by simply indicating the fact that it is accounted for in a given analysis.

The dependent variable captures the relationship between agricultural performance and land size (see section S6 for additional information on interpretation of the relationship). Different relationships can be found in the literature: (i) The performance indicator diminishes as the size of the holding increases, indicating an IR, and (ii) the performance indicator improves as the size of the land increases, showing a direct relationship. A third category shows a nonmonotonic relationship between performance and land size across all farm sizes at a given moment in time: IR may be recorded for the smallest farms but then weakens and eventually inverses, evolving into a “U-shaped” relationship, as in recent examples from China (*59*), Colombia (*60*), India (*27*), Kenya (*53*, *61*), Malawi (*62*), the Philippines (*63*), Tajikistan (*64*), and Zambia (*65*). Given their contribution to the debate, nonmonotonic relationships are recorded as part of the evidence gathered. However, since those relationships are a combination of IR and direct relationships, our main analysis does not include nonmonotonic as an alternative to IR (however, an exploration through multinomial logit is presented in data file S1). It focuses instead on modeling mutually exclusive relationships, i.e., the probability of detecting IR versus the probability of detecting a direct relationship. A fourth category comprises statistically nonsignificant estimates, showing positive, negative, or nonmonotonic relationships between land area and performance.

To explain the potential relationship, a number of independent variables are included as proxies of reasons pointed out in the literature. First, the type of indicator used in the analysis is taken into consideration. The wide variety of indicators used to capture the relationship between land size and agricultural performance was aggregated into three categories. The first group, labeled as “gross output,” includes indicators related to yields or total output (in physical or monetary units), which are by far the most frequently used indicators in the literature (*7*, *9*, *35*). The econometric studies using these indicators, which represent 66% of our sample, assumed that farm size only affects the production/revenues of the farm. The other two groups of indicators not only consider that land size affects production/revenues but also input costs or use in the econometric relationship. Those indicators assessing the (total or partial) profitability of agricultural land are grouped into a category called “net value” (14% of the sample), while those assessing the technical efficiency or total factor productivity of agricultural holdings are placed in the category labeled “efficiency” (20% of the sample) (see classification and definitions in section S7).

To account for the heterogeneity of cases and identify key drivers of the relationship, four groups of control variables were used in the meta-regression model, namely, (i) bibliometric characteristics of the publication (i.e., specialized literature); (ii) methodological dimensions, including the use of control variables discussed in the literature (GPS measurement of land area, soil quality and/or slope, access to irrigation, access to credit, use of mechanical and/or animal power, off-farm activity, and use of panel data); (iii) characteristics of not only the sample studied in each case, such as average land size, but also the most recent year of study and the type of data source (LSMS-ISA, national statistics, or ad hoc surveys); and, last, (iv) main crop grown, as identified in the paper, and location of the land in a warm arid or semi-arid area. See section S8 for a detailed description of the variables.

### Analysis

We estimate a logit model to examine the prevalence of IR in the literature and its drivers, distinguishing between management levels of households: farm (full unit) or plot (subunit). In some cases, data from the same country were gathered from different sources, so the observations cannot be considered completely independent. To cope with potential correlation between error terms across land performance results within a particular country, SEs are grouped by country cluster. Hence, the model assumes that observations are independent across countries but not necessarily within each country (see section S9 for clustering).

Meta-regressions may be vulnerable to publication bias, in that significant results are more likely to be published than nonsignificant ones (*58*, *66*–*68*). To control for this publication bias, alongside significant results on the relationship between land size and agricultural performance, nonsignificant estimates were also recorded, allowing expansion of the analysis. In addition, the strategy for collecting data (i.e., papers) allows us to control a second potential publication bias, based on the nature of the material available (specialized bias). The approach gathered cases both from specialized publications focusing on the role of land size in performance and from general studies that accounted for land size in their analysis but had no any specific interest in the relationship (i.e., it was not the core question of the paper). These complementary sources allow us to control the meta-regression procedure in case the specific literature tends to be directed toward a given type of relationship between land size and agricultural performance. In addition, potential differences between cross section and matched panel data studies were explored with a dummy variable capturing this characteristic, explicitly classifying as panel data those cases that handled several periods implementing methods for controlling time-invariant unobservable characteristics (e.g., fixed effects).

In addition to the controls mentioned above, the issue of publication quality is addressed using weights. The weighted approach is used here to qualify information based on the quality of the publication. Hence, more weight is given to cases published in scientific papers in journals ranked in the top two quartiles (Q1 and Q2) for impact. The rest of the information is weighted according to the relevance of the journal (Q3 and Q4) or to the other two publication categories included in the study (book chapters and working papers) (see section S10 for detailed information about weights).

## RESULTS

### Distribution of available material

**Fig. 1:**
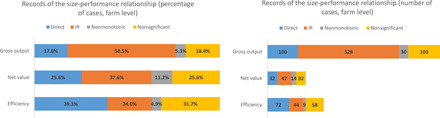
Farm-level land size–performance relationship, by performance indicator group, by percentage and total number of cases.

As illustrated in Fig. 1, IR between farm area and agricultural performance is more frequently recorded when the relationship is assessed through gross output indicators (e.g., yield or total production). Thus, we found IR in 58% of total cases gathered from the literature. However, the less commonly used, but more economically informative, indicators do not point to this prevalence of IR. When using net value indicators, there are no substantial differences in the profitability of small and larger farms; in the case of efficiency indicators, the relationship swaps: Larger farms tend to be more efficient than their smaller counterparts (39%).

Besides the prevalence of IR or direct relationship, nonmonotonic (e.g., U-shaped) relationships are recorded in a proportion of cases, as illustrated in Fig. 1. A similar overview is provided when looking at the data from the analyses at plot level, although, here, there is a smaller sample (please refer to data file S1).

The analysis of the relationship between land size and agricultural performance concentrates on certain regions and countries (SSA: Ethiopia, Nigeria, and Uganda; Asia: Bangladesh, India, and China). Our records for cases in LAC and MENA indicate a significantly weaker exploration of the relationship over the past two decades.

### Are small farms more productive than larger ones? What does the literature say?

A meta-regression is conducted using a weighted logit model at the different management levels. The weighted logit model fits the maximum likelihood function, represented by the following expression

$$\text{ln}L=\Sigma_{j\in S}w_{j}\text{ln}F(x_{j}\beta )+\Sigma_{j\notin S}w_{j}\text{ln}\{1-F(x_{j}\beta )\}$$ (1)

where *S* is the set of observations *j* such that *y_j_* ≠ 0; *y_j_* is a dichotomous-dependent variable, taking the value 1 when IR is recorded and 0 in the case of direct relationship; *x_j_* refers to the independent variables; β is a vector of regression coefficients; *F* is the cumulative logistic function *F*(*x_j_*β) = *e*^*x_j_*β^/(1 + *e*^*x_j_*β^); and *w_j_* represents the weights.

A number of models were run to assess the drivers of the probability of finding IR in the literature and to obtain robust estimates. BIC (Bayesian information criterion) and AIC (Akaike’s information criterion) goodness of fit were used to improve the logit models, when including not only the full set of control variables but also weights for quality, to the unweighted models. The decrease in both BIC and AIC exceeded 10, indicating “strong evidence” that the main models retained offer a significantly and substantively better fit with the data than do control models (i.e., limited control variables models and unweighted models). The specific values of these criteria are presented, along with the model estimates, in sections S11 and S12.

**Fig. 2:**
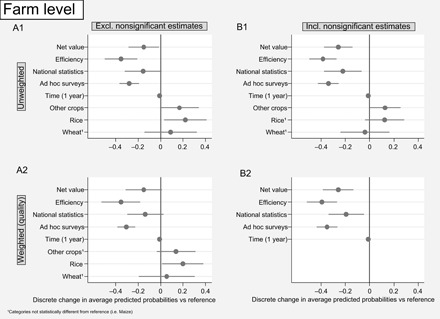
Statistically significant discrete change in predicted probabilities of IR, from base level (zero or any unit and category of reference) at farm level. (**A** and **B**) A1 model excluding nonsignificant category, unweighted. B1 model including nonsignificant category, unweighted. A2 model excluding nonsignificant category, weighted according to quality of publication. B2 model including nonsignificant category, weighted according to quality of publication. Introducing nonsignificant estimates (moving from A to B models) does not alter the overall trends. Introducing weights does not significantly alter the unweighted results either, as the direction and magnitude of probabilities remain very similar between 1 and 2 models. Both results (A versus B; 1 versus 2) suggest overall robustness of the approach. In all cases, the reference for net value and efficiency is gross output; for other crops, rice, and wheat, the reference is maize. Hence, according to model B2, a study assessing efficiency is 40% points less likely to record IR than one assessing gross output (65%). The full models are presented in the section S11, along with details on weighting criteria (section S10).

A first general observation from the estimates at farm level (Table 1) is that the performance indicator chosen is the main variable affecting the general direction of the relationship between agricultural performance and land size. This is regardless of the analysis method (unweighted versus weighted approach, whether including data from nonsignificant estimates), as can be seen in Fig. 2. This is valid for the analyses both at farm level and plot level, in comparable proportions (see Table 1). All things equal, applying gross output indicators to a given analysis at farm level yields a probability of finding IR of 65 to 77% (weighted approach). In contrast, this probability drops to 39 to 62% using net value indicators and further to 26 to 42% using efficiency analyses. Similar results are found when the analysis is conducted at a smaller unit management level (i.e., plot level). Figure 2 also shows that there are significant differences in the probability of recording IR when we compare net value and efficiency indicators with gross output (see sections S13 and S14 for additional testing of data).

**Table 1:** Probability of recording IR, by indicator group and management level, at two levels of analysis. Table gathering the probabilities of recording IR, according to performance indicators (gross output, net value, or efficiency) and management level (farm or plot), following a logit model. Two robustness checks are also introduced in the form of weights and enlarged sample. The enlarged sample involves including nonsignificant estimates recorded in the sample studied as an alternative to IR, in addition to statistically significant direct relationships initially recorded. For example, it is possible for farm-level analyses to increase from 498 to 648 cases by not only restricting the analysis to IR versus direct relationship but also to any other possibility (with the exclusion of nonmonotonic for analytical reasons). Models also account for the quality of the publications considered in the case of weighted models. Full models, along with weighting criteria, are developed in sections S11 and S12.

	**Unweighted**				**Weighted (quality of publication)**			
	**Farm**		**Plot**		**Farm**		**Plot**	
	**Excluding** **nonsignificant**	**Including** **nonsignificant**	**Excluding** **nonsignificant**	**Including** **nonsignificant**	**Excluding** **nonsignificant**	**Including** **nonsignificant**	**Excluding** **nonsignificant**	**Including** **nonsignificant**
**Gross output**	0.778	0.655	0.789	0.644	0.768	0.650	0.783	0.625
**Net value**	0.629	0.397	0.586	0.333	0.619	0.391	0.606	0.357
**Efficiency**	0.426	0.271	0.364	0.236	0.415	0.257	0.376	0.222
***n***	*498*	*648*	*190*	*247*	*498*	*648*	*190*	*247*

Considering the other independent variables, the type of data source has a significant influence on the probability of finding IR at farm level, regardless of type of analysis (weighted versus unweighted, whether including nonsignificant estimates) (see Fig. 2). Thus, we are less likely to find IR when the analysis is based on data from national statistics or ad hoc surveys than when using LSMS-ISA data. These results link with the question of limited land size range and potentially invalidate a large share of the existing evidence as policy (*43*). The larger the sample farm size range, the greater the possibility of capturing a direct relationship or a change in the relationship from IR to a direct relationship, i.e., a nonmonotonic relationship (*53*, *69*). Specifically designed surveys to represent smallholders (e.g., LSMS-ISA) are more associated with IR than ad hoc ones or national statistics, which may have different, wider sampling strategies (see Table 2 for a descriptive analysis of the data source). This effect wanes when the analysis is performed at plot level, since the range in plot sizes is more homogeneous among different data sources.

**Table 2:** Farm size distribution by data source*. Table showing the distribution of the average farm size by data source. Mean shows the mean of the average farm size considered in the original analysis, and min/max shows the minimum/maximum average of the farm size. The smallest range of average farm sizes corresponds to LSMS-ISA data, whereas the largest range corresponds to national statistics.

**Data source**	***n***	**Mean**	**SD**	**Min**	**Max**
**LSMS-ISA**	109	4.177	4.644	0.206	20.260
**National statistics**	138	18.055	128.561	0.169	1074
**Ad hoc**	450	5.010	19.389	0.028	280.900

*Brazil is excluded, as being an outlier.

When looking at the main crop of the farm, models show a larger probability of finding IR when the farm is cultivated with rice than with maize. The highest prevalence of IR on rice than on maize farms may be explained by the different adoption rates of improved technologies (i.e., packages of improved seeds and fertilizers) between rice (mainly cultivated in Asia as also recorded by the literature collected) and maize (mainly cultivated in SSA, as shown in data file S1). About 82% of the area in Asia was planted to major crops using improved technologies (mainly for rice and wheat) by 1998 with a rapid adoption by small farms (*70*). These technologies were fertilizer-responsive, high-yielding varieties that greatly improved land productivity and allow keeping the advantage of being small (*55*). In contrast, the adoption of maize technologies based on fertilizer-responsive, high-yielding varieties remained low in SSA (45% on average by 2005) (*71*, *72*) and with considerable regional differences. In turn, adoption rates for maize technologies were reported to range from 33% in Eastern Africa to 60% in West and Central Africa for 2006–2007 (*73*). This low and irregular adoption may hamper the performance of small farms on maize cultivation. One of the reasons behind these different adoption rates between rice in Asia and maize in SSA is based on the variety of food systems found in SSA in contrast to what it happens on Asia (which is mainly based on wheat and rice), making more risky the adoption of a single high-yielding crop technology (*55*, *74*).

**Fig. 3:**
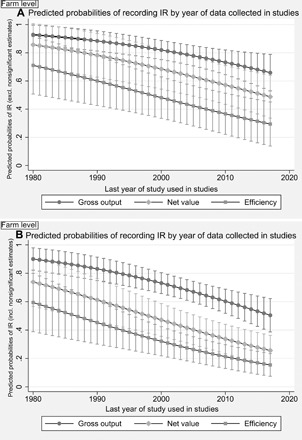
Predicted probabilities of recording IR, by the last year of study used in studies. When studies cover several years in pooled estimations, the last year of the series is recorded and used here. Farm level, weighted models. (**A**) Model A excluding nonsignificant category; (**B**) Model B including nonsignificant category. Early studies (data from 1990) show a higher probability of recording IR than the most recent ones. Gross output probabilities are significantly different between 1990/2000 and later years. For example, model B records over 80% probability of finding IR in the early 1990s, dropping to ~50% for 2017. The same applies to net value and efficiency between the older and newer datasets: Average probabilities of recording IR decrease with time. The models are presented in section S11.

**Fig. 4:**
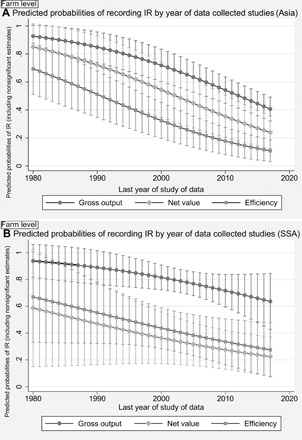
Predicted probabilities of recording IR in Asia and in SSA, by the last year of study used in studies. When studies cover several years in pooled estimations, the last year of the series is recorded and used here. Farm level, weighted models including nonsignificant records. (**A**) Model A Asia data; (**B**) model B SSA data. Early studies show a higher probability of recording IR than the most recent ones.

Another aspect to highlight is that studies into farm size–agricultural performance relationship using older data are more likely to record IR than those developed with more recent data. The most recent year of data used in a study is significantly associated with recording less IR, in any given analysis (Fig. 3). For illustration, the probability of recording IR using gross output indicators drops from 88% in 1990 to 66% in 2017 (from 83 to 50%, when considering nonsignificant estimates; see Fig. 3B). Although we can see some differences in the values of predicted probabilities depending on whether nonsignificant estimates are considered or excluded from the analysis, both models are very consistent in showing that the probability of recording IR is decreasing over time. The reasons behind the weakening of IR across time depend on the region analyzed (see Fig. 4).

As shown for many countries in Asia, increasing real agricultural wages over the course of those past two decades, along with absorptions of labor into nonagricultural sectors, induced substitution of labor by machine, mainly through machine rental or service providers [for the Philippines (*75*, *76*), Indonesia (*77*), Vietnam (*37*, *78*), China (*79*), India (*28*), and Bangladesh (*38*)]. As a result, the productivity advantage of smaller farms (IR) diminished and, in some cases, even reversed, increasing operational farm size among farmers through land rental markets as well. By contrast, in SSA agricultural intensification by use of improved technologies and mechanization has remained lower than expected in the light of population pressures over land and market access conditions (*80*, *81*). In addition, and compared to other developing regions, in SSA, most income in rural areas still derives from on-farm activities, and engagement of residents in nonagricultural wage employment is low (*82*–*84*). Consequently, the situation is still not comparable to the Asian case and substitution of labor by machinery is not happening on a large scale (*85*), weakening the IR but still definitively to a lesser extent. The different evolution of IR across time, more evident for Asia when looking at gross output indicators, can be seen in Fig. 4.

Looking at the literature analyzing the farm size–agricultural performance relationship (specialized versus generalist), results show that estimations from specialized literature are indistinguishable from those from general agricultural economics, rejecting a potential publication bias. However, there is a statistically significant difference within the literature dealing with plot-level analysis, where the specialized literature is more likely to record IR (see section S12 and fig. S3). Looking at the proportion of specialized papers at farm level (22.5%) or plot level (25%), there is no significant difference between the volumes of publications analyzing this topic at both types of management levels. The specialized bias remains at plot level even when controlling for quality of publications and conventional publication bias (including nonsignificant estimates) (see section S12 and fig. S3).

Last, the inclusion of variables looking at the methodological specification of the models (GPS measurement of land area, soil quality, access to irrigation, access to credit, and use of mechanical and/or animal power) does not seem to have any influence on the probability of finding IR at farm level. However, if the analysis is focused on studies using the most frequently analyzed indicator (i.e., gross output), then we can see that variables such as GPS measurement of the farm and soil quality do play an important role in the probability of finding IR (see a detailed analysis at gross output level in section S13). More concretely, the inclusion of soil quality negatively influences the probability of recording IR when the analysis is focused on the gross output indicator at farm level. This quality variable may be related just to the features of the soil or also to the knowledge of the farmers on managing their small holdings (*44*–*47*).

When the analysis is performed at plot level, we can perceive that the specification of the model is somewhat more important than at farm level, and accounting for soil quality negatively influences the probability of recording IR (see section S12 and fig. S4). This is somehow expected since at plot level, the quality of the soil is more homogeneous, and controlling for this variable may be crucial for agricultural performance. Farm level includes several plots, and a bad soil quality plot may be compensated by another with good soil quality.

Although about a 10th of all cases considered in the analysis qualified as matching panel data, the tests conducted did not highlight a difference between good quality panel data studies and other types of analyses. The possible explanation is that although quality panel data tend to depress IR prevalence, they do so in matter of degree. Here, the alternative to IR is dichotomic; hence, controlling for unobservable characteristics may reduce the severity of IR recording but not necessarily reject it, implying a direct relationship (see section S11 for details).

## DISCUSSION

This paper is the first comprehensive attempt to map the literature on the land size–performance relationship and to identify potential drivers in developing economies. Results demonstrate that, far from being an established “fact” in development economics, IR cannot be taken for granted because this relationship is highly dependent on the performance indicator used to assess agricultural performance. It is highly probable to find IR in the literature if the analysis is based on gross output indicators, but this likelihood drops when the analysis uses net value or efficiency indicators. Our results are proved to be robust, since they do not depend on the specific literature analyzing the topic (specialized literature bias) or on considering only significant results published in the literature (publication bias).

The prevalence of IR has frequently been explained by the intensive use of family labor resulting in higher crop yields (gross output) but with low profitability or input use efficiency (*15*, *25*, *30*, *86*). Despite the importance of labor productivity in explaining differences between large and small farms (*35*, *43*), this indicator is not directly included in the study as a singled out indicator group, given the limited disaggregated cases found in the literature, preventing robust analysis. However, labor productivity is indirectly included in the review, through net value (profitability) and efficiency indicators. In terms of the latter indicators, smallholders appear less efficient because of low labor productivity, usually due to difficulties in incorporating capital (e.g., animal or mechanical power) in their small farms.

The fundamental consideration of labor productivity, alongside the use of other inputs, by net value and efficiency indicators, indicates that any meaningful assessment of the land size–performance relationship should be driven by either profitability or efficiency differentials, instead of the misplaced importance given to only yields, total output, or value equivalent.

The potential role of accounting for off-farm activities/income was also explored and tested but indicated that the prevalence of recording IR over alternative relationships is not particularly sensitive to this specification. This is the case for the main models encompassing all studies and indicators. However, further analysis looking at the subsample of gross output studies shows that a study accounting for off-farm activity/income is less likely to report IR and statistically significantly so. Moreover, the overall performance of the model is improved by this addition, as indicated by the AIC and BIC indicators. This points not only to the probable greater sensitivity of gross output analyses to misspecification but also to the importance of labor market functioning as key explanatory factor to the prevalence of IR (*8*, *34*, *71*), inter alia (details of both estimations are available in section S7 in data file S1).

Engaging in the discussion on gross output indicators, some authors also pointed out that farm size may not even be considered a key determinant of yields (*35*) and that this suggests a myopic view of yield gains, as specificities of the land such as soil quality or location-specific factors (access to markets, extension services, etc.) are stronger drivers of performance. However, this study did not find any significant correlation between the inclusion of these factors in the analysis and the probability of finding IR at farm level when encompassing all performance indicators. This correlation does, however, exist at plot level, or when focusing on gross output indicators at farm level (see sections S12 and S13). Including the slope and/or soil quality reduces the probability of finding IR by 10% when focusing on gross output indicators (i.e., using gross output model) or by 14% when the analysis is conducted at plot level. This is in line with parts of the literature (*44*, *46*–*48*), showing that IR weakens when these soil and slope characteristics are considered in the analysis.

The environmental dimension of agricultural performance is not included in the analysis. A dedicated review is warranted to assess the differences in performance of smallholdings compared to larger farms in terms of natural resource and energy efficiency, greenhouse gas (GHG) emissions, soil conservation, or pollution prevention. Despite the paucity of relevant data on direct environmental indicators, the control for the number of crops or intercropping diversity could provide some clues as to the sustainability of a given type of relationship prevailing (i.e., IR, direct, or other). However, the level of detail in most of the studies prevents any meaningful analysis, given that they seldom record this information.

In addition to being dependent on the type of performance indicator used in the literature to assess agricultural performance, the results of the study show that IR may not be considered universal for any crop or at any time. When tested over time, the severity of IR has been reported as declining (*28*, *36*–*39*). However, the potentially gradual evolution of the relationship cannot be captured by this meta-regression, because the approach is based on the direction of the relationship as categorical variables (IR versus direct). Given the great diversity of performance indicators and estimation methods, the analysis does not consider the grade of IR, i.e., whether it is severe or “light” (close to becoming a direct relationship). Only clear-cut differences are robustly demonstrated, and the model does capture a decrease in the probability of recording IR over the analyzed period. Assuming that our specification captures the most critical factors and improvements in estimation (i.e., improved techniques and availability and quality of data), this trend may be associated with a process of change in rural labor markets. In the case of countries that are relatively more land abundant, it could even be capturing signs of structural transformation of agriculture and indications of a growing number of medium-scale farms (*54*, *87*) in these countries.

Last, results also show that the recording of IR may have been altered by the impact of the data source on the detection of IR. Since part of the research conducted is based on population surveys, which are clearly focused on smallholders (i.e., no records for medium or larger farms), it is very likely that alternative relationships were not correctly assessed (direct or nonmonotonic, i.e., U-shaped). This result is in line with (*35*, *43*, *52*), reinforcing the necessity of expanding current surveys to better address the representation of medium and large farms in developing countries, especially in relatively land-abundant ones.

Future directions include widening the framework of performance from a productive/economic dimension to a sustainability one, by including environmental and social aspects. The environmental dimension can initially be explored by reinforcing a detailed description of activities and crops, starting, for example, with the average number of crops per land unit (i.e., basic agrobiodiversity assessment). Energy use, soil loss, and GHG emissions data would ideally then be considered in such an environmental assessment. Next steps to assess the sustainability of farms would involve the social pillar, considering for instance the dynamics of the labor force used in agriculture and its repercussions on the community. Moreover and given that smallholders will remain the most important share of rural households (*34*, *88*, *89*), specifically testing poverty and food and nutrition security indicators performance against the size of farms could provide relevant orientation for targeted support programs.

### Some implications for development

Focusing development aid intervention on agriculture remains a valid recommendation, based on its advantage in terms of multiplier effects over other sectors of the economy, especially in SSA (*90*–*92*). Among other drivers, development policies are influenced by the idea that small farms produce more per hectare than larger ones and, thus, advocate advancement in agriculture to be driven by smallholders. Increasing gross output productivity can make a difference in terms of poverty reduction under high population density contexts (*81*) and for the most marginal farmers with limited or no access to off-farm or migration opportunities (*55*). Although output growth partially responds to the needs of rural development, the associated output indicators often entail a simplistic linkage between agricultural output increase and improvement in the disposable income of smallholder farming households. Evidence for SSA shows that successful strategies to improve food security are only dependent on agricultural output increases in the type of limited contexts mentioned above (*93*).

In the short run, any support to this type of smallholders is fully justified both as social and agricultural sector development program. However, when considering the long run agricultural development strategy, factor market access (e.g., labor, land, credit, and insurance) shall be key as they have been identified as more determinant factors for poverty alleviation and food security improvements (*93*, *94*), beyond simple agricultural output increases.

In this sense, development policy must consider the results offered by economic indicators (e.g., net value or efficiency) as policy drivers in the long run. These indicators show not only the factor use by farmers but also the potential substitution among them, pointing to the best scale of holdings in a given current markets situation and/or the market conditions to promote for improving farm performance, independently of their size. On the basis of the results of the study these indicators show the potential for favoring the development of somewhat larger farms than the currently existing ones by, for instance, promoting long-term land rental markets enabling medium-sized farms in land-abundant countries (*54*, *87*).

## Supplementary Material

abb8235_Data_file_S1.xlsx

abb8235_Data_file_S2.dta

abb8235_SM.pdf

abb8235_Data_file_S3.do

## SUPPLEMENTARY MATERIALS

Supplementary Materials for this article is available at http://advances.sciencemag.org/cgi/content/full/6/41/eabb8235/DC1
