# Supplementary material for: Are small farms more performant than larger ones in developing countries?
Source: Sci Adv. 2020 Oct 9;6(41):eabb8235. doi: 10.1126/sciadv.abb8235 (PMC7546703; doi:10.1126/sciadv.abb8235)
Supplement: abb8235_SM.pdf [file abb8235_SM.pdf]

Science Advances
AAAS

advances.sciencemag.org/cgi/content/full/6/41/eabb8235/DC1

Supplementary Materials for

Are small farms more performant than larger ones in developing countries?

P. A. Garzón Delvaux*, L. Riesgo, S. Gomez y Paloma

*Corresponding author. Email: p_a_garzon@yahoo.co.uk

Published 9 October 2020, *Sci. Adv.* **6**, eabb8235 (2020)
DOI: 10.1126/sciadv.abb8235

The PDF file includes:

Supplementary Materials and Methods
Tables S1 to S8
Figs. S1 to S4
References

Other Supplementary Material for this manuscript includes the following:

(available at advances.sciencemag.org/cgi/content/full/6/41/eabb8235/DC1)

Data files S1 to S3

Materials and Methods

S1: From papers to selected cases

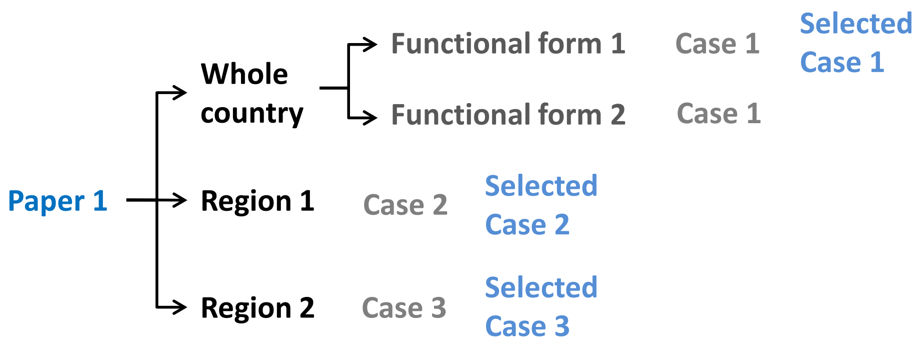

Fig. S 1. Sampling and analysis units

The unit of analysis is that of observations or cases within each paper. A single paper may illustrate various cases (various countries, national and regional estimates, or various crops, etc.). In addition, a single paper may use various approaches (i.e. functional forms) to assess the effect of the land size on the performance indicator. Only the selected cases are included in the analysis, hence controlling for undue replication by including cases which are considering the same original sample but with approaches of varying quality. Fig. S1 illustrates a scenario with three cases that would have been selected for the analysis: those corresponding to the two regions and the whole country analysis having used the method identified by the author(s) of the paper as the most appropriate to the analysis (here 'functional form 1').

S2: Table with full inclusion and exclusion criteria

Table S 1. Inclusion and exclusion criteria

	Inclusion	Exclusion
**Type of publication**	Peer-reviewed papers, published books, working papers of selected organisations or papers from snowball identification.	Unpublished material, PhD theses in repositories, working papers not meeting the inclusion criteria, conference papers.
**Years of publication**	Papers published from 1997 to 2018 (both years included).	Publication dating from 1996 or before, and after 2018. No exclusion criteria for year of data used in publication. Key publications are referred to in the introduction and feed the interpretation, though.
**Nature of analysis**	Regression analyses. Any econometric approach with a performance indicator as dependant variable and at least land size as independent variable.	Descriptive or narrative studies, commentaries, experiments, trials, simulation or model, summaries or meta-analysis (this last type was used for snowball purposes, however).
**Data**	Survey or census data at household / farm /plot/ parcel level.	Aggregated data beyond household or farm data (e.g. data of size at regional, national or supranational levels).

	Inclusion	Exclusion
**Scope**	Crop farm, Mixed farms, crop parcels/plots.	Cattle, dairy, aquatic or animal husbandry production systems (e.g. pure pastoral systems)
**Performance indicators***	Gross Output (total production, total revenue, yield, value per area), Net Value (ratio revenues/cost, total profit, gross margin, net farm income, profit per area, gross margin per area, net farm income per area), Efficiency (technical, economic, etc. and TFP)	Performance indicator on differentials (e.g. gap on productivity)
**Explanatory variables (at least)**	plot / farm / parcel / area / land size	
**Populations**	Developing countries, including most BRICS	Developed countries, including Russia, Armenia, Belarus, Moldavia, Taiwan, South Korea, Mauritius, West Indian European or American dominions
**Languages**	English, French, Spanish, Portuguese, Italian	Other languages (e.g. Mandarin, Japanese or Hindi)

* Environmental indicators using as proxy of the adoption of soil and water conservation measures, including tree planting were explored in the screening of the literature. Given the heterogeneity, sparse evidence of actual environmental performance, they were excluded from the analysis. Labor productivity indicators were also excluded from the analysis due to their low representation in the literature.

S3: Identification and data gathering process

A total of 474 papers were retained from a pool of 1,658 identified papers, extracted from a search where over 118,000 titles were initially scanned (1997-2018), as illustrated by **¡Error! No se encuentra el origen de la referencia.**, following the guidelines of PRISMA (see S4: Search strategy and keywords). In turn this currently translates into 1,135 selected cases or observations.

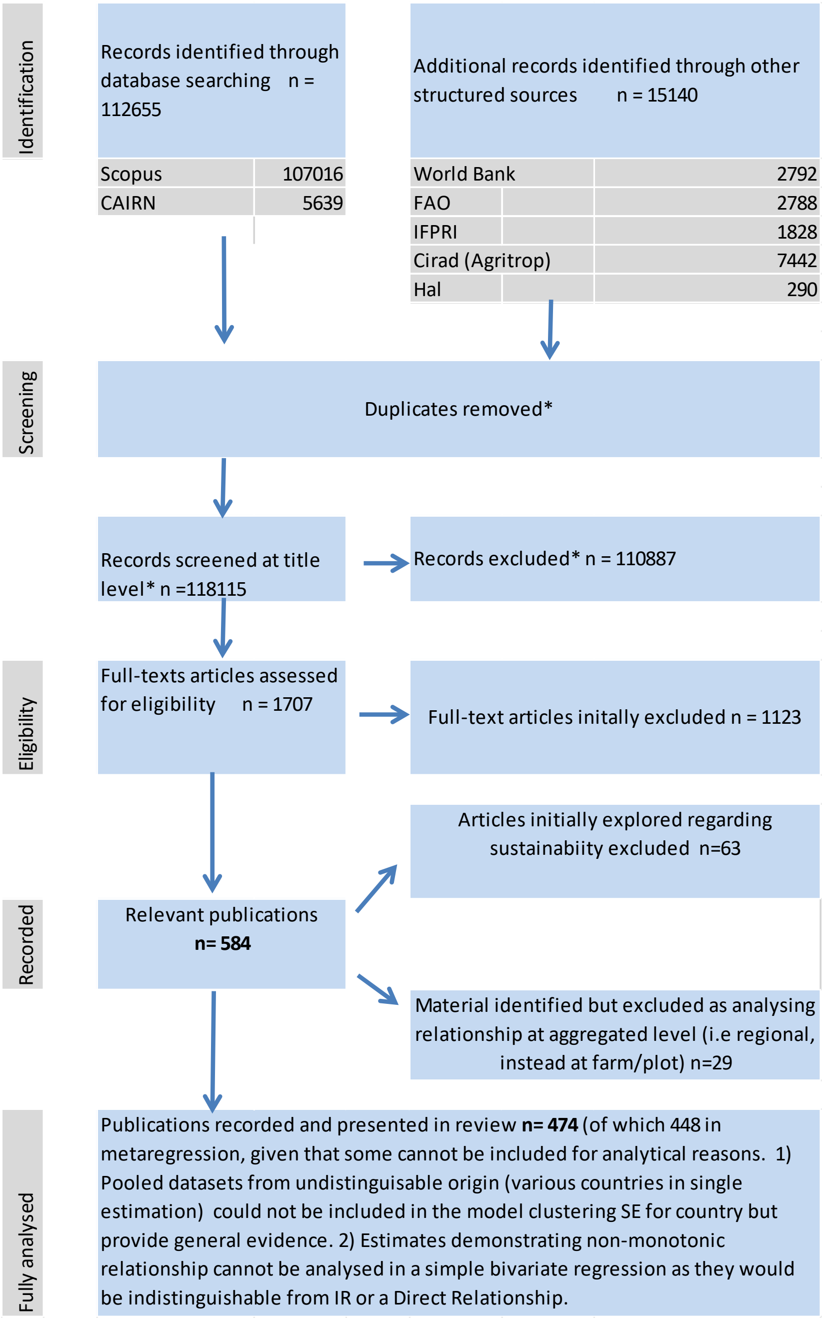

* Indicative only, given that some databases could only be manually explored, webpage by webpage, preventing the download and subsequent record of full lists to identify duplicates.

Fig. S 2. Flow diagram illustrating papers identified and selected, following full text assessment. Diagram stages are adapted from the PRISM PRISMA/ PRISMA-P guidance checklists

S4: Search strategy and keywords

Keywords

The initial scoping exercise consisted in identifying relevant search terms for both the specialized literature and the broader literature. The result of this initial set of search terms was the basis for the construction of the main library of relevant references.

Initial successive searches in SCOPUS are the result of the following combination of keywords:

Box S1. Combination of keywords for searching material in SCOPUS*

**"inverse relationship"**	AND	"agriculture"						
**"size productivity relationship"**								
**"yields"**	AND	"agriculture"	AND	"land"	AND	"size"	OR	"area"
**"farm"**	AND	"productivity"						
**"Ricardian"**	AND	"climate change"						
**"net crop revenue"**								
**"performance"**	AND	"farm"						
**"performance"**	AND	"agriculture"						

**"efficiency"**	AND	"agriculture"	
*<br>**"environmental indicator"**	AND	"agriculture"	*Also included as part of the original search. Not pursed in the final analysis.*

Based on the combination of keywords showed in Box 1, three final sets of search strings were used for SCOPUS, the main source for both the specialized and broader literature. Such search strings were identified conducting a test against known key references from the first search including the following reference authors: Barrett, C.B.; Benjamin, D.; Carletto, G.; Collier, P; Dercon, S., Deininger, K.; Gollin, D.; Helfand, S.M.; Jayne, T.; Lamb, R.L.; Otsuka, K.; Rozelle, S.

Regarding the ***first set*** of the main terms, the following combination of keywords was introduced:

> *"agricult*" OR "farm" OR "plot" OR "parcel" OR "area" OR "land" AND "inverse" AND "relationship"*

The **second set** of main terms includes the following keywords:

> *"agricult*" OR "farm" OR "plot" OR "parcel" OR "area" OR "land"*

The search was also focused on the different **type of indicators** that can be found in the literature, as the following:

> *"revenue" OR "income" OR "yield" OR "value" OR "efficiency" OR "productivity" OR "production" OR "profit" OR "rent" OR "performance"*

In addition, some **approach** terms were included to identify different methodologies of analyses:

> *"production function" OR "ricardian" OR "stochastic" OR "frontier" OR "DEA" OR "regression"*

In opposition to the precise geographical classification of documents that can be found in institutional databases (e.g. World Bank, IFPRI), SCOPUS has a less functional metadata system[1]. To address this, two identical searches were

[1] Web of Science does not fare better, following testing.

conducted, first using the metadata and excluding the irrelevant countries[2] and secondly including in the search string the individual names of countries in English (see below).

The results from the successive initial searches and the definitive search strings were merged so to have a more effective geographical coverage of the sources.

The search exploring the institutional databases in English used the following terms successively assuming that institutional repositories perform better with simpler search strings, as can be seen in Box 2.

Box S2. Combination of keywords for searching material in institutional databases

**"land"**	AND	"size"	OR	"area"	OR	"plot"	OR	"parcel"	OR	"surface"
**"yields"**										
**"inverse relationship"**										
**"farm productivity"**										
**"ricardian"**	AND	"climate change"								

The organizational databases offered the possibility to also select with precision the region from the metadata of documents; hence the focus of the searches was restricted to the macro region of interest or individual countries when possible.

In turn, the search exploring French databases and institutions was constructed, using the terms specified in Box 3.

[2] For the metadata on geographical location, a simple search with "agri*" was generated to produce the most complete list of options before engaging with the search string of interest.

Box S3. Combination of keywords for searching material in French

**"taille "**	ET	"exploitation agricole"								
**"relation inverse"**	ET	"agri*"								
**"Cobb Douglas"**	OU	"frontière de production"	OU	"fonction de production"	OU	"efficacité technique"	OU	"rendements"	ET	"agri*"
**"productivité "**	ET	"terre"								
**"ricardien"**	ET	" changement climatique "								

When needed, a string of the individual names of the countries of interest was also included in the search in French. The analysis of papers identified included English, French, Spanish, Portuguese and Italian literature.

List of countries and macro region searched in English and French, as introduced in search engines.

English

"Antigua and Barbuda" OR aruba OR bahamas OR barbados OR "Cayman Islands" OR cuba OR dominica OR "Dominican Republic" OR grenada OR guadeloupe OR haiti OR jamaica OR martinique OR "Puerto Rico" OR "Saint Barthélemy" OR "Saint Kitts and Nevis" OR "Saint Lucia" OR "Saint Vincent and the Grenadines" OR "Trinidad and Tobago" OR "Turks and Caicos Islands" OR "Virgin Islands" OR belize OR "Costa Rica" OR "El Salvador" OR guatemala OR honduras OR mexico OR nicaragua OR panama OR argentina OR bolivia OR brazil OR chile OR colombia OR ecuador OR "French Guiana" OR guyana OR paraguay OR peru OR suriname OR uruguay OR venezuela OR lac OR "Latin America and the Caribbean" OR caribbean OR "Latin America" OR "Central America"

OR "South America" OR antilles OR "West Indies" OR angola OR benin OR botswana OR "Burkina Faso" OR burundi OR "Cabo Verde" OR cameroon OR "Central African Republic" OR car OR rca OR rdc OR chad OR comoros OR congo OR "Cote d'Ivoire" OR "ivory coast" OR djibouti OR eritrea OR ethiopia OR gabon OR gambia OR ghana OR guinea OR kenya OR lesotho OR liberia OR madagascar OR malawi OR mali OR mauritania OR mozambique OR namibia OR niger OR nigeria OR rwanda OR "Sao Tome and Principe" OR "Sao Tome e Principe" OR senegal OR seychelles OR "Sierra Leone" OR somalia OR "South Africa" OR sudan OR swaziland OR tanzania OR togo OR uganda OR zambia OR zimbabwe OR afrique OR africa OR sahel OR algeria OR bahrain OR egypt OR iran OR iraq OR israel OR jordan OR kuwait OR lebanon OR libya OR morocco OR oman OR qatar OR "Saudi Arabia" OR syria OR tunisia OR "United Arab Emirates" OR uae OR yemen OR maroc OR mena OR "North Africa" OR "middle east" OR "middle-east" OR "near-east" OR iran OR iraq OR palestine OR qatar OR syria OR turkey OR kazakhstan OR kyrgyzstan OR tajikistan OR turkmenistan OR uzbekistan OR china OR mongolia OR tibet OR singapore OR philippines OR afghanistan OR bangladesh OR bhutan OR india OR maldives OR nepal OR pakistan OR "Sri Lanka" OR bahrain OR brunei OR cambodia OR indonesia OR laos OR malaysia OR burma OR myanmar OR thailand OR "Timor-Leste" OR vietnam OR fiji OR kiribati OR "Marshall Islands" OR micronesia OR nauru OR palau OR "Papua New Guinea" OR samoa OR "Solomon Islands" OR tonga OR tuvalu OR vanuatu OR asia OR pacific))

French

Afghanistan OU "Afrique du Sud" OU Albanie OU Algérie OU Angola OU Anguilla OU "Antigua-et-Barbuda" OU "Arabie Saoudite" OU Argentine OU Azerbaïdjan OU Bahamas OU Bahreïn OU Bangladesh OU Barbade OU Belize OU Bénin OU Bermudes OU Bhoutan OU Birmanie OU Myanmar OU Bolivie OU Botswana OU Brésil OU Brunei OU "Burkina Faso" OU Burundi Cambodge OU Cameroun OU "Cap-Vert" OU Chili OU Chine OU Colombie OU Comores OU "Corée du Nord" OU "Corée du Sud" OU "Costa Rica" OU "Côte d'Ivoire" OU Cuba OU Djibouti OU Dominique OU Égypte OU "Émirats Arabes Unis" OU Équateur OU Érythrée OU "États Fédérés de Micronésie" OU Éthiopie OU Fidji OU Gabon OU Gambie OU "Îles Sandwich du Sud" OU Ghana OU Grenade OU Guam OU Guatemala Guinée OU Guyan* OU Haïti Honduras OU "Hong-Kong" OU "Île Christmas" OU "Îles Caïmans" OU "Îles Cocos" OU Keeling OU" Îles Cook" OU "Îles Mariannes du Nord" OU "Îles Marshall" OU "Îles Pitcairn" OU "Îles Salomon" OU "Îles Turks et Caïques" OU "Îles Vierges" OU Inde OU Indonésie Iran OU Iraq OU Israël OU

Jamaïque OU Jordanie OU Kazakhstan OU Kenya OU Kirghizistan OU Kiribati OU Koweït OU Laos OU Lesotho OU Liban OU Libéria OU Libye OU Macao OU Madagascar OU Malaisie OU Malawi OU Maldives OU Mali OU Maroc OU Mauritanie OU Mayotte OU Mexique OU Moldavie OU Mongolie OU Montserrat OU Mozambique OU Namibie OU Nauru OU Népal OU Nicaragua OU Niger Nigéria OU Niué OU "Nouvelle-Calédonie" OU Oman OU Ouganda OU Ouzbékistan OU Pakistan OU Palaos OU Panama OU "Papouasie-Nouvelle-Guinée" OU Paraguay OU Pérou OU Philippines OU "Polynésie Française" OU Porto Rico OU Qatar OU "République Centrafricaine" OU Congo OU "République Dominicaine" OU Congo OU Rwanda OU "Sahara Occidental" OU "Saint-Kitts-et-Nevis" OU "Saint-Vincent-et-les Grenadines" OU "Sainte-Lucie" OU Salvador OU Samoa OU "Sao Tomé-et-Principe" OU Sénégal OU Seychelles OU "Sierra Leone" OU Singapour OU Somalie OU Soudan OU "Sri Lanka" OU Suriname OU Swaziland OU Syrie OU Tadjikistan OU Taïwan OU Tanzanie OU Tchad OU Thaïlande OU "Timor Oriental "OU Togo OU Tonga OU "Trinité-et-Tobago" OU Tunisie OU Turkménistan OU Turquie OU Tuvalu OU Uruguay OU Vanuatu OU Venezuela OU "Viet Nam" OU "Wallis et Futuna" OU Yémen OU Zambie OU Zimbabwe

S5: Synthetic references of papers included, by quartile, publication and author

Quartiles 1 and 2

Njikam, O.& Alhadji, H. A."Technical Efficiency among Smallholder Rice Farmers: A Comparative Analysis of Three Agro-ecological Zones in Cameroon". African Development Review. (2017)

Alene, A. D.& Hassan, R. M."The determinants of farm-level technical efficiency among adopters of improved maize production technology in western Ethiopia". Agrekon. (2003)

Aye, G. C.& Mungatana, E. D."Technological innovation and efficiency in the Nigerian maize sector: Parametric stochastic and non-parametric distance function approaches". Agrekon. (2011)

Bizimana, C. et al. "Farm size, land fragmentation and economic efficiency in Southern Rwanda". Agrekon. (2005)

Freguin-Gresh, S. et al. "Demythifying contract farming: evidence from rural South Africa". Agrekon. (2012)

Mushunje, A.& Belete, A."Efficiency of Zimbabwean small scale communal farmers". Agrekon. (2001)

Ngwenya, S. A. et al. "The relationship between farm size and the technical inefficiency of production of wheat farmers in the Eastern Free State, Province of South Africa". Agrekon. (1997)

Mishra, A. K. et al. "Cooperatives, contract farming and farm size: The case of tomato producers in Nepal". Agribusiness. (2018)

Jha, R."Some Imperatives of the Green Revolution: Technical Efficiency and Ownership of Inputs in Indian Agriculture". Agricultural and Resource Economics Review. (1999)

Abate, G. T. et al. "The impact of the use of new technologies on farmers wheat yield in Ethiopia: evidence from randomized controlled trial". Agricultural Economics. (2018)

Abdulai, A. N."Impact of conservation agriculture technology on household welfare in Zambia". Agricultural Economics. (2016)

Abrar, S.& Morrissey, O."Supply response in Ethiopia: accounting for technical inefficiency". Agricultural Economics. (2006)

Aguilar, A. et al. " Decomposition of gender differentials in agricultural productivity in Ethiopia". Agricultural Economics. (2015)

Alene, A. D.& Manyong, V. M."Farmer-to-farmer technology diffusion and yield variation among adopters: the case of improved cowpea in northern Nigeria". Agricultural Economics. (2006)

Ali, A. et al. "Impacts of tenancy arrangements on investment and efficiency: evidence from Pakistan". Agricultural Economics. (2012)

Ayenew, H. Y. et al. "Decent rural employment farm production efficiency: empirical evidence from Tanzania and Ethiopia". Agricultural Economics. (2017)

Bakhshoodeh, M.& Thomson, K. J."Input and output technical efficiencies of wheat production in Kerman, Iran". Agricultural Economics. (2001)

Birthal, P. S. et al. "The impacts of information on returns from farming: evidence from nationally representative farm survey in India". Agricultural Economics. (2015)

Boshrabadi, H. M. et al. "Technical efficiency and environmental-technological gaps in wheat production in Kerman province of Iran". Agricultural Economics. (2008)

Bozoglu, M.& Ceyhan, V."Measuring the technical efficiency and exploring the inefficiency determinants of vegetable farms in Samsun province, Turkey". Agricultural Economics. (2007)

deGraft-Johnson, M. et al. "On the transferability of the Asian rice green revolution to rainfed areas in sub-Saharan Africa: an assessment of technology intervention in Northern Ghana". Agricultural Economics. (2014)

Dey, M. M. et al. "The impact of integrated aquaculture–agriculture on small-scale farms in Southern Malawi". Agricultural Economics. (2010)

Foltz, J. D. "Credit market access and profitability in Tunisian agriculture". Agricultural Economics. (2004)

Fuwa, N. et al. "Are small-scale rice farmers in Eastern India really inefficient? Examining the effects of microtopography on technical efficiency estimates". Agricultural Economics. (2007)

Govereh, J.& Jayne, T. S."Cash cropping and food crop productivity: synergies or trade-offs?". Agricultural Economics. (2003)

Guirkinger, C.& Boucher, S. R."Credit constraints and productivity in Peruvian agriculture". Agricultural Economics. (2008)

Hazarika, G.& Alwang, J."Access to credit, plot size and cost inefficiency among smallholder tobacco cultivators in Malawi". Agricultural Economics. (2003)

Helfand, S. M.& Levine, E. S."Farm size and the determinants of productive efficiency in the Brazilian Center-West". Agricultural Economics. (2004)

Huang, Y.& Kalirajan, K. P."Potential of China's grain production: evidence from the household data". Agricultural Economics. (1997)

Karamba, R. W.& Winters, P. C."Gender and agricultural productivity: implications of the Farm Input Subsidy Program in Malawi". Agricultural Economics. (2015)

Kimhi, A."Plot size and maize productivity in Zambia: is there an inverse relationship?". Agricultural Economics. (2006)

Larson, D. F. et al. " Should African rural development strategies depend on smallholder farms? An exploration of the inverse-productivity hypothesis". Agricultural Economics. (2014)

Pattanayak, S.& Mercer, D. E."Valuing soil conservation benefits of agroforestry: Contour hedgerows in the Eastern Visayas, Philippines". Agricultural Economics. (1998)

Pender, J. et al. "Strategies to increase agricultural productivity and reduce land degradation: evidence from Uganda". Agricultural Economics. (2004)

Rahman, S."Environmental impacts of technological change in Bangladesh agriculture: farmers' perceptions, determinants, and effects on resource allocation decisions". Agricultural Economics. (2005)

Satriawan, E.& Swinton, S. M."Does human capital raise farm or nonfarm earning more? New insight from a rural Pakistan household panel". Agricultural Economics. (2007)

Sauer, J.& Mendoza-Escalante, A."Poor but allocatively efficient—evidence from the Eastern Amazon". Agricultural Economics. (2007)

Savastano, S.& Scandizzo, P. L."Optimal farm size in an uncertain land market: the case of Kyrgyz Republic". Agricultural Economics. (2009)

Seymour, G."Women's empowerment in agriculture: implications for technical efficiency in rural Bangladesh". Agricultural Economics. (2017)

Slavchevska, V."Gender differences in agricultural productivity: the case of Tanzania". Agricultural Economics. (2015)

Stifel, D.& Minten, B."Isolation and agricultural productivity". Agricultural Economics. (2008)

Tadesse, B.& Krishnamoorthy, S."Technical efficiency in paddy farms of Tamil Nadu: An analysis based on farm size and ecological zone". Agricultural Economics. (1997)

Takeshima, H."Custom-hired tractor services and returns to scale in smallholder agriculture: a production function approach". Agricultural Economics. (2017)

Tipraqsa, P.& Schreinemachers, P."Agricultural commercialization of Karen Hill tribes in northern Thailand". Agricultural Economics. (2009)

Townsend, R. F. et al. "Farm size, productivity and returns to scale in agriculture revisited: a case study of wine producers in South Africa". Agricultural Economics. (1998)

Wang, J. et al. "The impact of climate change on China's agriculture". Agricultural Economics. (2009)

Yorobe, J. M. et al. "Yield and income effects of rice varieties with tolerance of multiple abiotic stresses: the case of green super rice (GSR) and flooding in the Philippines". Agricultural Economics. (2016)

Khan, H.& Ali, F."Measurement of productive efficiency of tomato growers in Peshawar, Pakistan". Agricultural Economics Czech. (2013)

Onumah, J. A. et al. "Meta-frontier analysis of organic and conventional cocoa production in Ghana". Agricultural Economics Czech. (2013)

Shrestha, R. B. et al. "Efficiency of small scale vegetable farms: Policy implications for the rural poverty reduction in Nepal". Agricultural Economics Czech. (2016)

Belloumi, M.& Matoussi, M. S. "A stochastic frontier approach for measuring technical efficiencies of date farms in southern Tunisia". Agricultural Economics Research Review. (2006)

Sekhon, M. K. et al. "Technical efficiency in crop production: a region-wise analysis". Agricultural Economics Research Review. (2010)

Abdallah, A."Agricultural credit and technical efficiency in Ghana: is there a nexus?". Agricultural Finance Review. (2016)

Akudugu, M. A"Agricultural productivity, credit and farm size nexus in Africa: a case study of Ghana". Agricultural Finance Review. (2016)

Coulibaly, J. Y. et al. "Adoption of agroforestry and the impact on household food security among farmers in Malawi". Agricultural Systems. (2017)

Krishna, V. V. & Veettil, P. C."Productivity and efficiency impacts of conservation tillage in northwest Indo-Gangetic Plains". Agricultural Systems. (2014)

Lu, H. et al. "Assessing the impacts of land fragmentation and plot size on yields and costs: a translog production model and cost function approach". Agricultural Systems. (2018)

Ndlovu, P. V. et al. "Productivity and efficiency analysis of maize under conservation agriculture in Zimbabwe". Agricultural Systems. (2014)

Rahman, S.& Parkinson, R. J."Productivity and soil fertility relationships in rice production systems". Agricultural Systems. (2007)

Yigezu, Y. A. et al. "Implications of a shift in irrigation technology on resource use efficiency: A Syrian case". Agricultural Systems. (2013)

Frija, A. et al. "Water use and technical efficiencies in horticultural greenhouses in Tunisia". Agricultural Water Management. (2009)

Zhou, Y. et al. "Economic impacts on farm households due to water reallocation in China's Chaobai watershed". Agricultural Water Management. (2009)

Wongnaa, C. A.& Awunyo-Vitor, D."Achieving sustainable development goals on no poverty and zero hunger: does technical efficiency of Ghana's maize farmers matter?". Agriculture and Food Security. (2018)

Mariano, M. J. et al. "Are irrigated farming ecosystems more productive than rainfed farming systems in rice production in the Philippines?". Agriculture, Ecosystems and Environment. (2010)

Adegbite, O.& Adeoye, I. B."Technical efficiency of pineapple production in Osun State, Nigeria". Agris on-line papers in Economics and Informatics. (2015)

Prochazka, P. et al. "Some factors affecting the efficiency of potato production, under Al-Ghab plain conditions, Syrian Arab Republic". Agronomy Research. (2017)

Poungchompu, S.& Chantanop, S."Factor affecting technical efficiency of smallholder rubber farming in Northeast Thailand". American Journal of Agricultural and Biological Sciences. (2015)

Arslan, A.& Taylor, J. E."Farmers' Subjective Valuation of Subsistence Crops: The Case of Traditional Maize in Mexico". American Journal of Agricultural Economics. (2009)

Assunção, J. J.& Braido, L. H. B. "Testing household-specific explanations for the inverse productivity relationship". American Journal of Agricultural Economics. (2007)

Barrett, C. B. et al. "Better Technology, Better Plots, or Better Farmers? Identifying Changes in Productivity and Risk among Malagasy Rice Farmers". American Journal of Agricultural Economics. (2004)

Deininger, K. et al. "Does sharecropping affect long-term investment? Evidence from west bengal's tenancy reforms". American Journal of Agricultural Economics. (2013)

Hayes, J. et al. " Tenure Security, Investment and Productivity in Gambian Agriculture: A Generalized Probit Analysis". American Journal of Agricultural Economics. (1997)

Le, K. T."Shadow Wages and Shadow Income in Farmers' Labor Supply Functions". American Journal of Agricultural Economics. (2009)

Le, K. T."Separation Hypothesis Tests in the Agricultural Household Model". American Journal of Agricultural Economics. (2010)

Marenya, P. P. & Barrett, C. B."State-conditional fertilizer yield response on Western Kenyan Farms". American Journal of Agricultural Economics. (2009)

Rao, E. J. O. et al. "Farmer participation in supermarket channels, production technology and efficiency: the case of vegetables in Kenya". American Journal of Agricultural Economics. (2012)

Yang, D. T."Education in production : Measuring labor quality and management". American Journal of Agricultural Economics. (1997)

Athipanyakul, T. et al. "Key factors for improving technical efficiency of upland rice production". American Journal of Applied Sciences. (2014)

Elias, A. et al. "The effect of agricultural extension service on the technical efficiency of teff (Eragrostistef) producers in Ethiopia". American Journal of Applied Sciences. (2014)

Huq, A. S. M. A.& Arshad, F. M."Technical efficiency of Chili production". American Journal of Applied Sciences. (2010)

Idris, N. D. M. H. et al. "Determinants of Technical Efficiency on Pineapple Farming". American Journal of Applied Sciences. (2013)

Supaporn, P."Determinants of technical efficiency of sugarcane production among small holder farmers in Lao PDR". American Journal of Applied Sciences. (2015)

Mishra, A. K. et al. "Production risks, risk preference and contract farming: impact on food security in India". Applied Economic Perspectives and Policy. (2018)

Adhikari, C. B.& Bjorndal, T."Analyses of technical efficiency using SDF and DEA models: evidence from Nepalese agriculture". Applied Economics. (2012)

Asadullah, M. N.& Rahman, S."Farm productivity and efficiency in rural Bangladesh: the role of education revisited". Applied Economics. (2009)

Salim, R.& Hossain, A."Market deregulation, trade liberalization and productive efficiency in Bangladesh agriculture: an empirical analysis". Applied Economics. (2006)

Shankar, B. et al. "Production risk, pesticide use and GM crop technology in South Africa". Applied Economics. (2008)

Villano, R.& Fleming, E."Technical inefficiency and production risk in rice farming: evidence from Central Luzon Philippines". Asian Economic Journal. (2006)

Watto, M. A.& Mugera, A. W."Measuring production and irrigation efficiencies of rice farms: evidence from the Punjab province, Pakistan". Asian Economic Journal. (2014)

Kousar, R.& Abdulai, A."Off-farm work, land tenancy contracts and investments in soil conservation measures in rural Pakistan". Australian Journal of Agricultural and Resource Economics. (2015)

Ma, W. et al. "Farm machinery use, off-farm employment and farm performance in China". Australian Journal of Agricultural and Resource Economics. (2018)

Benjamin, D.& Brandt, L."Property rights, labour markets, and efficiency in a transition economy: the case of rural China". Canadian Journal of Economics/Revue canadienne d'économique. (2002)

Zhang, L. et al. "How Off-farm Employment Affects Technical Efficiency of China's Farms: The Case of Jiangsu". China & World Economy. (2016)

Holly Wang, H. et al. "Is contract farming a risk management instrument for Chinese farmers?: Evidence from a survey of vegetable farmers in Shandong". China Agricultural Economic Review. (2011)

Li, G. et al. "Re-examining the inverse relationship between farm size and efficiency: the empirical evidence in China". China Agricultural Economic Review. (2013)

Tang, L. et al. "Do agricultural services contribute to cost saving? Evidence from Chinese farmers". China Agricultural Economic Review. (2018)

Wang, Jx. et al. "Impacts of climate change on net crop revenue in North and South China". China Agricultural Economic Review. (2014)

Chen, Z. et al. "Farm technology and technical efficiency: evidence from four regions in China". China Economic Review. (2009)

Feng, S. et al. "Land rental market, off-farm employment and agricultural production in Southeast China: A plot-level case study". China Economic Review. (2010)

Wu, Z. et al. "Land consolidation and productivity in chinese household crop production". China Economic Review. (2005)

Zhang, X. et al. "Mechanization outsourcing clusters and division of labor in Chinese agriculture". China Economic Review. (2017)

de Brauw, A. et al. "feminization of agriculture in China? Myths surrounding women's participation in farming". China Quarterly. (2008)

Closset, M. et al. "Measuring the economic impact of climate change on agriculture: a Ricardian analysis of farmlands in Tajikistan". Climate and Development. (2015)

Khanal, U. et al. "Autonomous adaptations to climate change and rice productivity: a case study of the Tanahum district, Nepal". Climate and Development. (2018)

Sadiq, S. et al. "Ricardian analysis of climate change-agriculture linkages in Pakistan". Climate and Development. (2018)

Gunathilaka, R. P. D. et al. "The impact of changing climate on perennial crops: the case of tea production in Sri Lanka". Climatic Change. (2017)

Khanal, U. et al. "Climate change adaptation strategies and food productivity in Nepal: a counterfactual analysis". Climatic Change. (2018)

Kurukulasuriya, P.& Ajwad, M. I."Application of the Ricardian Technique to estimate the impact of climate change on smallholder farming in Sri Lanka". Climatic Change. (2007)

Chen, Z. et al. "Inverse relationship between productivity and farm size: the case of China". Contemporary Economic Policy. (2011)

Kipkoech, A. K. et al. "Assessing yield and efficiency implications of relying on parasitoids for control of cereal stemborers: The case of small-scale maize farmers in Kenya". Crop Protection. (2008)

Baha, M. R."Does plot size matter? Evidence from maize production in Babati District, Tanzania". Development in Practice. (2016)

Berazneva, J. et al. "Allocation and valuation of smallholder maize residues in Western Kenya". Ecological Economics. (2018)

Mahadevan, R."The high price of sweetness: the twin challenges of efficiency and soil erosion in Fiji's sugar industry". Ecological Economics. (2008)

Pascual, U."Land use intensification potential in slash-and-burn farming through improvements in technical efficiency". Ecological Economics. (2005)

Useche, P.& Blare, T."Traditional vs. modern production systems: Price and nonmarket considerations of cacao producers in Northern Ecuador". Ecological Economics. (2013)

Croppenstedt, A.& Muller, C."The Impact of Farmers' Health and Nutritional Status on Their Productivity and Efficiency: Evidence from Ethiopia". Economic Development and Cultural Change. (2000)

Huang, J. et al. "Genetically modified rice, yields and pesticides: assessing farm-level productivity effects in China". Economic Development and Cultural Change. (2008)

Owens, T. et al. "The Impact of Agricultural Extension on Farm Production in Resettlement Areas of Zimbabwe". Economic Development and Cultural Change. (2003)

Tu, V. H. et al. "Technical and environmental efficiency of eco-friendly rice production in the upstream region of the Vietnamese Mekong delta". Environment Development and Sustainability. (2018)

Abdulai, A.& Abdulai, A."Examining the impact of conservation agriculture on environmental efficiency among maize farmers in Zambia". Environment and Development Economics. (2016)

Abdulai, A.& Binder, C. R."Slash-and-burn cultivation practice and agricultural input demand and output supply". Environment and Development Economics. (2006)

Ahmed, M. M. et al. "Measurement and sources of technical efficiency of land tenure contracts in Ethiopia". Environment and Development Economics. (2002)

Gorst, A. et al. "Crop productivity and adaptation to climate change in Pakistan". Environment and Development Economics. (2018)

Ma, X. et al. "Land tenure security and technical efficiency: new insights from a case study in Northwestern China". Environment and Development Economics. (2017)

Mendelsohn, R. et al. "A Ricardian analysis of Mexican farms". Environment and Development Economics. (2009)

Otsuka, K. et al. "Land tenure and the management of land and trees: the case of customary land tenure areas of Ghana". Environment and Development Economics. (2003)

Qaim, M.& De Janvry, A."Bt cotton and pesticide use in Argentina: economic and environmental effects". Environment and Development Economics. (2005)

Mullan, K. et al. "Converting Forests to Farms: The Economic Benefits of Clearing Forests in Agricultural Settlements in the Amazon". Environmental and Resource Economics. (2017)

Reyes, T. et al. "Socio-economic comparison between traditional and improved cultivation methods in agroforestry systems, East Usambara Mountains, Tanzania". Environmental Management. (2005)

Ullah, A.& Perret, S. R."Technical- and Environmental-Efficiency Analysis of Irrigated Cotton-Cropping Systems in Punjab, Pakistan Using Data Envelopment Analysis". Environmental Management. (2014)

Veeck, G. et al. "The Modern Chinese Farmer: Technical Adoptions and Marketing Innovations among Vegetable Farmers in Shijiazhuang, Hebei". Eurasian Geography and Economics. (2003)

Deininger, K.& Jin, S."Tenure security and land-related investment: Evidence from Ethiopia". European Economic Review. (2006)

Deininger, K. et al. "Efficiency and equity impacts of rural land rental restrictions: evidence from India". European Economic Review. (2008)

Paresys, L. et al. "Feeding the world while reducing farmer poverty? Analysis of rice relative yield and labour productivity gaps in two Beninese villages". European Journal of Agronomy. (2018)

Hailu, G. et al. "Determinants of the productivity of teff in Ethiopia". European Journal of Development Research. (2016)

Lee, Y. H."A stochastic production frontier model with group-specific temporal variation in technical efficiency". European Journal of Operational Research. (2006)

Gupta, A. et al. "Is technology change good for cotton farmers? A local-economy analysis from the Tanzania Lake Zone". European Review of Agricultural Economics. (2018)

Blanc, E. et al. "Determinants of crop yield and profit of family farms: evidence from the Senegal River valley". Experimental Agriculture. (2016)

Duguma, L. A. et al. "The financial return of cereal farming for smallholder farmers in the central highlands of Ethiopia". Experimental Agriculture. (2010)

Jote, A. et al. "Assessing the efficiency of sweet potato producers in the southern region of Ethiopia". Experimental Agriculture. (2017)

wa Githinji, M. et al. "Small and productive: Kenyan women and crop choice". Feminist Economics. (2014)

Berre, D. et al. "Thinking beyond agronomic yield gap: Smallholder farm efficiency under contrasted livelihood strategies in Malawi". Field Crops Research. (2017)

Binam, J. N. et al. "Factors affecting the technical efficiency among smallholder farmers in the slash and burn agriculture zone of Cameroon". Food Policy. (2004)

de Janvry, A.& Sadoulet, E."Rural poverty in latin America Determinants and exit paths". Food Policy. (2000)

Headey, D. et al. "Land constraints and agricultural intensification in Ethiopia: a village-level analysis of high-potential areas". Food Policy. (2014)

Liverpool-Tasie, L. S. O. et al. "Is increasing fertilizer use for maize production in SSA a profitable position? Evidence from Nigeria". Food Policy. (2017)

Ma, W.& Abdulai, A."Does cooperative membership improve household welfare? Evidence from apple farmers in China". Food Policy. (2016)

Mishra, A. K. et al. "How can organic rice be a boon to smallholders? Evidence from contract farming in India". Food Policy. (2018)

Sheahan, M. et al. "Are Kenyan farmers under-utilizing fertilizer? Implications for input intensification strategies and research". Food Policy. (2013)

Takeshima, H. et al. "Effects of tractor ownership on returns-to-scale in agriculture: evidence from maize in Ghana". Food Policy. (2018)

Vellema, W. et al. "The effect of specialty coffee certification on household livelihood strategies and specialisation". Food Policy. (2015)

Wollni, M.& Brümmer, B."Productive efficiency of specialty and conventional coffee farmers in Costa Rica: Accounting for technological heterogeneity and self-selection". Food Policy. (2012)

Diagne, M. et al. "Self-sufficiency policy and irrigated rice productivity in the Senegal River Valley". Food Security. (2013)

Keil, A. et al. "Zero-tillage as a pathway for sustainable wheat intensification in the Eastern Indo-Gangetic Plains: does it work in farmers' fields?". Food Security. (2015)

Ogwuike, P et al. "Weed management in upland rice in sub-Saharan Africa: impact on labor and crop productivity". Food Security. (2014)

Ragasa, C.& Chapoto, A."Moving in the right direction? The role of price subsidies in fertilizer use and maize productivity in Ghana". Food Security. (2017)

Rios, A. R.& Shively, G. E."FARM SIZE, IRRIGATION INFRASTRUCTURE, AND THE EFFICIENCY OF COFFEE PRODUCTION IN VIETNAM". Forests, trees and livelihoods. (2015)

Kabubo-Mariara, J.& Karanja, F. K."The economic impact of climate change on`` Kenyan crop agriculture: A Ricardian approach". Global and Planetary Change. (2007)

Athukorala, W.& Wilson, C."Groundwater overuse and farm-level technical efficiency: evidence from Sri Lanka". Hydrogeology Journal. (2012)

Narayanamoorthy, A."Farmer's education and productivity of crops: a new approach". Indian Journal of Agricultural Economics. (2000)

Reddy, AR& Sen, C"Technical inefficiency in rice production and its relationship with farm-specific socio-economic characteristics". Indian Journal of Agricultural Economics. (2004)

Sharma, H. R.& Sharma, R. K."Farm size-productivity relationship: Empirical evidence from an agriculturally developed region of Himachal Pradesh". Indian Journal of Agricultural Economics. (2000)

Ajayi, C. O.& Olutumise, A. I."Determinants of food security and technical efficiency of cassava farmers in Ondo State, Nigeria ". International Food and Agribusiness Management Review. (2018)

Jara-Rojas, R. et al. "Technical efficiency and marketing channels among small-scale farmers: evidence for raspberry production in Chile". International Food and Agribusiness Management Review. (2018)

Fonta, W. M. et al. "A Ricardian valuation of the impact of climate change on Nigerian cocoa production. Insight for adaptation policy". International Journal of Climate Change Strategies and Management. (2018)

Hossain, M. S. et al. "Climate change and crop farming in Bangladesh: an analysis of economic impacts". International Journal of Climate Change Strategies and Management. (2018)

Ahmed, M. N. & Schmitz, P. M."Climate change impacts and the value of adaptation - can crop adjustments help farmers in Pakistan?". International Journal of Global Warming. (2015)

Mariyono, J."Productivity growth of Indonesian rice production: sources and efforts to improve performance". International Journal of Productivity and Performance Management. (2018)

Alene, A. D.& Hassan, R. M."The Efficiency of Traditional and Hybrid Maize Production in Eastern Ethiopia: An Extended Efficiency Decomposition Approach". Journal of African Economies. (2005)

Asfaw, S. et al. "Agricultural technology adoption under climate change in the Sahel: Micro-evidence from Niger". Journal of African Economies. (2016)

Carletto, C. et al. "From guesstimates to GPStimates: land area measurement and implications for agricultural analysis". Journal of African Economies. (2015)

Cunguara, B.& Moder, K."Is Agricultural Extension Helping the Poor? Evidence from Rural Mozambique". Journal of African Economies. (2011)

Haji, J."Production efficiency of smallholders' vegetable-dominated mixed farming system in eastern Ethiopia: A non-parametric approach". Journal of African Economies. (2007)

Nerman, M."Households' income-generating activities and marginal returns to labour in rural Tanzania". Journal of African Economies. (2015)

Fletschner, D.& Zepeda, L."Efficiency of Small Landholders in Eastern Paraguay". Journal of Agricultural and Resource Economics. (2002)

Abedullah et al. "Bt Cotton, Pesticide Use and Environmental Efficiency in Pakistan". Journal of Agricultural Economics. (2015)

Coelli, T. et al. "Technical, Allocative, Cost and Scale Efficiencies in Bangladesh Rice Cultivation: A Non-parametric Approach". Journal of Agricultural Economics. (2002)

Crost, B. et al. "Bias from farmer self-selection in genetically modified crop productivity estimates: evidence from Indian data". Journal of Agricultural Economics. (2007)

de Mey, Y. et al. "Estimating Bird Damage to Rice in Africa: Evidence from the Senegal River Valley". Journal of Agricultural Economics. (2012)

Henderson, H."Considering Technical and Allocative Efficiency in the Inverse Farm Size–Productivity Relationship". Journal of Agricultural Economics. (2015)

Holden, S. et al. "Market Imperfections and Land Productivity in the Ethiopian Highlands". Journal of Agricultural Economics. (2001)

Liverpool-Tasie, L. S. O."Is fertiliser use inconsistent with expected profit maximization in sub-Saharan Africa? Evidence from Nigeria". Journal of Agricultural Economics. (2017)

Ma, X. et al. "A question of integrity: variants of Bt cotton, pesticides and productivity in Pakistan". Journal of Agricultural Economics. (2017)

Michler, J. D.& Shively, G. E."Land Tenure, Tenure Security and Farm Efficiency: Panel Evidence from the Philippines". Journal of Agricultural Economics. (2015)

Rahman, S. et al. "Production efficiency of Jasmine rice producers in Northern and North-eastern Thailand". Journal of Agricultural Economics. (2009)

Solís, D. et al. "Technical efficiency among peasant farmers participating in natural resource management programmes in central America". Journal of Agricultural Economics. (2009)

Theriault, V.& Serra, R."Institutional environment and technical efficiency: a stochastic frontier analysis of cotton producers in West Africa". Journal of Agricultural Economics. (2014)

Verschelde, M. et al. " Challenging Small-Scale Farming: A Non-Parametric Analysis of the (Inverse) Relationship Between Farm Productivity and Farm Size in Burundi". Journal of Agricultural Economics. (2013)

Jin, S.& Deininger, K."Land rental markets in the process of rural structural transformation: productivity and equity impacts from China". Journal of comparative economics. (2009)

Liu, Z.& Zhuang, J."Determinants of technical efficiency in post-collective Chinese agriculture: evidence from farm-level data". Journal of comparative economics. (2000)

Abdulai, A. et al. "Land tenure differences and investment in land improvement measures: Theoretical and empirical analyses". Journal of Development Economics. (2011)

Cao, K. H.& Birchenall, J. A. "Agricultural productivity, structural change and economic growth in post-reform China". Journal of Development Economics. (2013)

Carletto, C. et al. " Fact or artifact: The impact of measurement errors on the farm size–productivity relationship". Journal of Development Economics. (2013)

Desiere, S& Jolliffe, D."Land productivity and plot size: Is measurement error driving the inverse relationship?". Journal of Development Economics. (2018)

Jolliffe, D."The impact of education in rural Ghana: examining household labor allocation and returns on and off the farm.". Journal of Development Economics. (2004)

Lamb, R. L."Inverse productivity: land quality, labor markets, and measurement error". Journal of Development Economics. (2003)

Larson, D. F.& Plessmann, F."Do farmers choose to be inefficient? Evidence from Bicol". Journal of Development Economics. (2009)

Sherlund, S. M. et al. "Smallholder technical efficiency controlling for environmental production conditions". Journal of Development Economics. (2002)

Dorward, A." Farm size and productivity in Malawian smallholder agriculture". Journal of Development Studies. (1999)

Fletschner, D. et al. "Risk, credit constraints and financial efficiency in Peruvian agriculture". Journal of Development Studies. (2010)

Kagin, J. et al. "Inverse Productivity or Inverse Efficiency? Evidence from Mexico". Journal of Development Studies. (2016)

Palacios-López, A.& López, R."The gender gap in agricultural productivity: the role of market imperfections". Journal of Development Studies. (2015)

Wouterse, F."The Role of Empowerment in Agricultural Production: Evidence from Rural Households in Niger". Journal of Development Studies. (2017)

Deininger, K.& Castagnini, R."Incidence and impact of land conflict in Uganda". Journal of Economic Behaviour and Organisation. (2006)

Mahadevan, R."The viability of Fiji's sugar industry". Journal of economic studies. (2009)

Musafiri, I.& Sjölander, P."The importance of off-farm employment for smallholder farmers in Rwanda". Journal of Economic Studies. (2018)

Selejio, O. et al. "Smallholder agricultural production efficiency of adopters and nonadopters of land conservation technologies in Tanzania". Journal of Environment and Development. (2018)

Rahman, S.& Hasan, M. K."Impact of environmental production conditions on productivity and efficiency: A case study of wheat farmers in Bangladesh". Journal of Environmental Management. (2008)

Karunarathna, M.& Wilson, C."Agricultural biodiversity and farm level technical efficiency: An empirical investigation". Journal of Forest Economics. (2017)

Akamin, A. et al. "efficiency and productivity analysis of vegetable farming within root and tuber-based systems in the humid tropics of Cameroon". Journal of Integrative Agriculture. (2017)

Abrar, S. et al. " Aggregate agricultural supply response in Ethiopia: a farm-level analysis.". Journal of International Development . (2004)

Chowdhury, N. T."The Relative Efficiency of Hired and Family Labour in Bangladesh Agriculture". Journal of International Development . (2016)

Mahadevan, R."The less than sweet solution to Fiji's sugar industry problems". Journal of International Development . (2009)

Murtaza, G.& Thapa, G. B."Factors affecting technical efficiency of small-scale apple farms in Balochistan Plateau, Pakistan". Journal of Mountain Science. (2017)

Zhang, S. et al. "Technical efficiency and its determinants of the various cropping systems in the purple-soiled, hilly region of southwestern China". Journal of Mountain Science. (2016)

Aravindakshan, S. et al. "Application of a bias-corrected meta-frontier approach and an endogenous switching regression to analyze the technical efficiency of conservation tillage for wheat in South Asia". Journal of productivity analysis. (2018)

Audibert, M."Technical Inefficiency Effects Among Paddy Farmers in the Villages of the ‘Office du Niger’, Mali, West Africa". Journal of Productivity Analysis. (1997)

Wouterse, F."Can human capital variables be technology changing? An empirical test for rural households in Burkina Faso". Journal of productivity analysis. (2016)

Yang, J. et al. "Migration, local off-farm employment, and agricultural production efficiency: evidence from China". Journal of Productivity Analysis. (2016)

Kamruzzaman, M. et al. "Evaluation of Economic Efficiency of Wheat Farms in a Region of Bangladesh under the Input Orientation Model". Journal of the Asia Pacific Economy. (2006)

Chandio, A. A. et al. "The Nexus of Agricultural Credit, Farm Size and Technical Efficiency in Sindh, Pakistan: A Stochastic Production Frontier Approach". Journal of the Saudi Society of Agricultural Sciences. (2018)

Huong, N. T. L. et al. "Economic impact of climate change on agriculture using Ricardian approach: A case of northwest Vietnam". Journal of the Saudi Society of Agricultural Sciences. (2018)

Koondhar, M. A. et al. "Comparing economic efficiency of wheat productivity in different cropping systems of Sindh province, Pakistan". Journal of the Saudi Society of Agricultural Sciences. (2018)

Külekçi, M."Technical efficiency analysis for oilseed sunflower farms: a case study in Erzurum, Turkey". Journal of the Science of Food and Agriculture. (2010)

Ali, D. A.& Deininger, K. "Is There a Farm Size–Productivity Relationship in African agriculture? Evidence from Rwanda". Land Economics. (2015)

Bellemare, M. F."The Productivity Impacts of Formal and Informal Land Rights: Evidence from Madagascar". Land Economics. (2013)

Deininger, K. et al. "Legal Knowledge and Economic Development: The Case of Land Rights in Uganda". Land Economics. (2008)

Deininger, K. et al. "Can labor market imperfections explain changes in the inverse farm size-productivity relationship?: Longitudinal evidence from rural India". Land Economics. (2018)

Jin, S.& Jayne, T. S."Land rental markets in Kenya: implications for efficiency, equity, household income, and poverty". Land Economics. (2013)

Kompas, Tom et al. "Productivity, Net Returns, and Efficiency: Land and Market Reform in Vietnamese Rice Production". Land Economics. (2012)

Linkow, B."Causes and Consequences of Perceived Land Tenure Insecurity: Survey Evidence from Burkina Faso". Land Economics. (2016)

Shively, G. E."Agricultural change, rural labor markets, and forest clearing: an illustrative case from the Philippines". Land Economics. (2001)

Atis, E."Economic impacts on cotton production due to land degradation in the Gediz Delta, Turkey". Land Use Policy. (2006)

Jiao, X. et al. "Rural household incomes and land grabbing in Cambodia". Land Use Policy. (2015)

Khanal, U. et al. "Farm performance analysis: technical efficiencies and technology gaps of Nepalese farmers in different agro-ecological regions". Land Use Policy. (2018)

Koirala, K. H. et al. "Impact of land ownership on productivity and efficiency of rice farmers: The case of the Philippines". Land Use Policy. (2016)

Manjunatha, A. V. et al. "Impact of land fragmentation, farm size, land ownership and crop diversity on profit and efficiency of irrigated farms in India". Land Use Policy. (2013)

Paes Herrera, G. et al. "Econometric analysis of income, productivity and diversification among smallholders in Brazil". Land Use Policy. (2018)

Ragasa, C.& Chapoto, A."Limits to Green Revolution in rice in Africa: the case of Ghana". Land Use Policy. (2017)

Rahman, S.& Rahman, M."Impact of land fragmentation and resource ownership on productivity and efficiency: the case of rice producers in Bangladesh". Land Use Policy. (2008)

Masud, M. M. et al. "Impact of climate change: an empirical investigation of Malaysian rice production". Mitigation and Adaptation Strategies for Global Change. (2014)

Bangwayo-Skeete, P. F. et al. "Crop biodiversity, productivity and production risk: Panel data micro-evidence from Ethiopia". Natural Resources Forum. (2012)

De los Santos-Montero, L. A. & Bravo-Ureta, B. E."Productivity effects and natural resource management: econometric evidence from POSAF-II in Nicaragua". Natural Resources Forum. (2017)

Kilic, O. et al. "Determinants of economic efficiency: A case study of hazelnut (Corylus avellana) farms in Samsun Province, Turkey". New Zealand Journal of Crop and Horticultural Science. (2009)

Afidchao, M. M. et al. "Analysing the farm level economic impact of GM corn in the Philippines". NJAS - Wageningen Journal of Life Sciences. (2014)

Banerjee, H. et al. "Understanding biophysical and socio-economic determinants of maize (Zea mays L.) yield variability in eastern India". NJAS - Wageningen Journal of Life Sciences. (2014)

Feng, S."Land rental, off-farm employment and technical efficiency of farm households in Jiangxi Province, China". NJAS - Wageningen Journal of Life Sciences. (2008)

Kansiime, M. K. et al. "Farm diversity and resource use efficiency: targeting agricultural policy interventions in East Africa farming systems". NJAS - Wageningen Journal of Life Sciences. (2018)

Binici, T. et al. "Assessing the efficiency of cotton production on the Harran Plain, Turkey". Outlook on Agriculture. (2006)

Tavva, S. et al. "Technical efficiency of wheat farmers and options for minimizing yield gaps in Afghanistan". Outlook on Agriculture. (2017)

Deininger, K.& Jin, S."Land Sales and Rental Markets in Transition: Evidence from Rural Vietnam". Oxford Bulletin of Economics and Statistics. (2008)

Gaurav, S.& Mishra, S."Farm Size and Returns to Cultivation in India: Revisiting an Old Debate". Oxford Development Studies. (2015)

Newell, A. et al. "Farm size and the intensity of land use in Gujarat". Oxford Economic Papers. (1997)

Arshad, M. et al. "Climate variability and yield risk in South Asia’s rice–wheat systems: Emerging evidence from Pakistan". Paddy and Water Environment. (2017)

Ebers, A. et al. "Production efficiency of rice farms in Thailand and Cambodia: a comparative analysis of Ubon Ratchathani and Stung Treng provinces". Paddy and Water Environment. (2017)

Kiatpathomchai, S. et al. "Investigating external effects of shrimp farming on rice farming in southern Thailand: a technical efficiency approach". Paddy and Water Environment. (2008)

Bakhsh, K. et al. "The economic role of livestock assets in cotton productivity in Punjab, Pakistan". Pakistan Journal of Agricultural Sciences. (2014)

Hussain, A. et al. "Technical Efficiency of Wheat Production in Rain-fed Areas: A Case Study of Punjab, Pakistan". Pakistan Journal of Agricultural Sciences. (2012)

Hussain, M. et al. "An econometric analysis of bed-furrow irrigation for cultivated wheat in irrigated areas of Punjab, Pakistan". Pakistan Journal of Agricultural Sciences. (2017)

Gatiso, T. T. et al. "The impact of Ebola virus disease (evd) epidemic on agricultural production and livelihoods in Liberia". PLOS Neglected Tropical Diseases. (2018)

Wu, Y. et al. "Policy distortions, farm size and the overuse of agricultural chemicals in China". PNAS. (2018)

Li, L.& Tonts, M."The impacts of temporary labour migration on farming systems of the Loess plateau, gansu province, China". Population, Space and Place. (2014)

Ferenji, B. T. & Heidhues, F."Study of total factor productivity of tef in Ethiopia: application of a growth decomposition approach". Quaterly Journal of International Agriculture. (2007)

Wang, Y.& Chen, X."Are farmers' adaptations enhancing food production? Evidence from China". Regional Environmental Change. (2018)

Guthiga, P. M. et al. "Does use of draft animal power increase economic efficiency of smallholder farms in Kenya?". Renewable Agriculture and Food Systems. (2007)

Murali, P.& Puthira Prathap, D."Technical Efficiency of Sugarcane Farms: An Econometric Analysis". Sugar Tech. (2017)

Imran, M. A. et al. "Impact of climate smart agriculture (CSA) practices on cotton production and livelihood of farmers in Punjab, Pakistan". Sustainability. (2018)

Nyuor, A. B. et al. "Economic impacts of climate change on cereal production: Implications for sustainable agriculture in Northern Ghana". Sustainability. (2016)

Roco, L. et al. "The impact of climatic change adaptation on agricultural productivity in Central Chile: a stochasctic production frontier approach". Sustainability. (2017)

Yang, Z. et al. "Investigating yield variability and inefficiency in rice production: a case study in central China". Sustainability. (2016)

Zeng, S. et al. "Assessing the impacts of land consolidation on agricultural technical efficiency of producers: A survey from Jiangsu province, China". Sustainability. (2018)

Aragón, F. M.& Rud, J. P."Polluting Industries and Agricultural Productivity: Evidence from Mining in Ghana". The Economic Journal. (2016)

Fafchamps, M.& Quisumbing, A. R."Human capital, productivity, and labor allocation in rural Pakistan". The Journal of Human Resources. (1999)

Paul, M.& wa Guithinji, M."Small farms, smaller plots: land size, fragmentation and productivity in Ethiopia". The Journal of Peasant Studies. (2018)

Ahmad, M.& Qureshi, S. K."Recent evidence on farm size and land productivity: implications for public policy". The Pakistan Development Review. (1999)

Larson, D. F.& Leon, M."How Endowments, Accumulations, and Choice Determine the Geography of Agricultural Productivity in Ecuador". The World Bank Economic Review. (2006)

Zhou, Q. et al. "Participatory irrigation management and irrigation water use efficiency in maize production: evidence from Zhangye city, northwestern China". Water. (2017)

Dai, X. et al. "Impact of agricultural water reallocation on crop yield and revenue: a case study in China". Water Policy. (2017)

Sharma, K. R. et al. "Stochastic frontier approach to measuring irrigation performance: An application to rice production under the two systems in the Tarai of Nepal". Water Resources Research. (2001)

Agarwal, B."Can group farms outperform individual family farms? Empirical insights from India". World Development. (2018)

Alene, A. D. et al. "Economic efficiency and supply response of women as farm managers: comparative evidence from Western Kenya". World Development. (2008)

Ali, D. A. et al. "Investigating the gender gap in agricultural productivity: evidence from Uganda". World Development. (2016)

Barrett, C. B. et al. " Reconsidering Conventional Explanations of the Inverse Productivity–Size Relationship". World Development. (2010)

Bolwig, S. et al. "The Economics of Smallholder Organic Contract Farming in Tropical Africa". World Development. (2009)

Deininger, K. et al. "Determinants and impacts of rural land market activity: evidence from Nicaragua". World Development. (2003)

Heltberg, R."Rural market imperfections and the farm size— productivity relationship: Evidence from Pakistan". World Development. (1998)

Kilic, T. et al. "Caught in a Productivity Trap: A Distributional Perspective on Gender Differences in Malawian Agriculture". World Development. (2015)

Kilic, T. et al. "Missing(ness) in Action. Selectivity bias in GPS-based land area measurements". World Development. (2013)

Markussen, T."Property rights, productivity, and common property resources: Insights from rural Cambodia". World Development. (2008)

Mochebelele, M. T. & Winter-Nelson, A."Migrant labor and farm technical efficiency in Lesotho". World Development. (2000)

Ragasa,C.& Mazunda, J."The Impact of agricultural extension services in the context of heavily subsidized input system: the case of Malawi". World Development. (2018)

Smith, R. E."Land tenure, fixed investment and farm productivity: evidence from Zambia's southern province". World Development. (2004)

Thirtle, C. et al. "Can gm technologies help the poor? The impact of GM cotton in Makhathini Flats, Kwazulu-natal". World Development. (2003)

Wouterse, F.& Taylor, J. E."Migration and Income Diversification:: Evidence from Burkina Faso". World Development. (2008)

Pedroso, R. et al. "Technical efficiency of rice production in the delta of the Vu Gia Thu Bon river basin, Central Vietnam". World Development Perspectives. (2018)

Quartiles 3 and 4

Thimothy, A. T.& Adeoti, A. I. "Gender inequalities and economic efficiency: new evidence from cassava-based farm holdings in rural South-western Nigeria". African Development Review. (2006)

Baiyegunhi, L. J. S.& Arnold, C. A."Economics of sugarcane production on large scale farms in the Eshowe/Entumeni areas of KwaZulu-Natal, South Africa". African Journal of Agricultural Research. (2011)

Saka, J. O.& Lawal, B. O."Determinants of adoption and productivity of improved rice varieties in southwestern Nigeria". African Journal of Biotechnology. (2009)

Vitale, J. et al. "The socio-economic impacts of gm cotton in Burkina Faso: does farm structure affect how benefits are distributed?". AgBioForum. (2016)

Ng'ombe, J. N."Technical efficiency of smallholder maize production in Zambia: a stochastic meta-frontier approach". Agrekon. (2017)

Ochieng, J. et al. "Commercialisation of food crops and farm productivity: evidence from smallholders in Central Africa". Agrekon. (2016)

Tijani, A. A. "Analysis of the technical efficiency of rice farms in Ijesha Land of Osun State, Nigeria". Agrekon. (2006)

Karimov, A. A."Factors affecting efficiency of cotton producers in rural Khorezm, Uzbekistan: re-examining the role of knowledge indicators in technical efficiency improvement". Agricultural and Food Economics. (2014)

Abdulai, A.& Regmi, P. P."Estimating labor supply of farm households under nonseparability: empirical evidence from Nepal". Agricultural Economics. (2000)

Ogundari, K."Resource-productivity, allocative efficiency and determinants of technical efficiency of rainfed rice farmers: A guide for food security policy in Nigeria". Agricultural Economics Czech. (2008)

Paudel, P.& Matsuoka, A."Cost efficiency estimates of maize production in Nepal: a case study of the Chitwan district". Agricultural Economics Czech. (2009)

Islam, K. M. Z. et al. "Do differences in technical efficiency explain the adoption rate of HYV rice? Evidence from Bangladesh". Agricultural Economics Review. (2012)

Haryanto, T. et al. "Technical efficiency and technology gap in Indonesian rice farming.". Agris on-line papers in Economics and Informatics. (2016)

Nguyen, B. H.& Yabe, M."Profit efficiency of tea production in the Northern mountainous region of Vietnam". Agris on-line papers in Economics and Informatics. (2015)

Ferdushi, K. et al. "Factors affecting rice farms in Bangladesh: a stochastic frontier approach". American-Eurasian Journal of Sustainable Agriculture. (2013)

Abate, G. T. et al. "Impact of agricultural cooperatives on smallholders' technical efficiency: empirical evidence from Ethiopia". Annals of Public and Cooperative Economics. (2014)

Wan, G. H.& Cheng, E."Effects of land fragmentation and returns to scale in the Chinese farming sector". Applied Economics. (2001)

Jha, R. et al. "Productivity, technical and allocative efficiency and farm size in wheat farming in India: a DEA approach". Applied Economics Letters. (2000)

Mariano, M. J. et al. "Technical efficiency of rice farms in different agroclimatic zones in the Philippines: an application of a stochastic metafrontier model". Asian Economic Journal. (2011)

Kerdsriserm, C. et al. "Comparative analysis of the technical efficiency of different production systems for rice farming in Eastern Thailand". Asian Journal of Scientific Research. (2018)

Wang, J. et al. "Is small still beautiful? A comparative study of rice farm size and productivity in China and India". China Agricultural Economic Review. (2015)

Abidoye, B. O. et al. "Structural Ricardian analysis of South-East Asian agriculture". Climate Change Economics. (2017)

Abidoye, B. O. et al. "South-East Asian Ricardian Studies Bangladesh, Sri Lanka, Thailand, and Vietnam". Climate Change Economics. (2017)

Freire, A. H. et al. "Economic efficiency of coffee in the south of minas gerais: One application of production frontier". Coffee Science. (2011)

Okoye, B. C. et al. "Differentials in technical efficiency among smallholder cassava farmers in Central Madagascar: A Cobb Douglas stochastic frontier production approach". Cogent Economics & Finance. (2016)

Mango, N. et al. "A stochastic frontier analysis of technical efficiency in smallholder maize production in Zimbabwe: the post-fast-track land reform outlook". Cogent Economics and Finance. (2015)

Binam, J. N. et al. "Technical efficiency and productivity potential of cocoa farmers in west African countries". Developing economies. (2008)

Bravo-Ureta, B. E.& Pinheiro, A. E."Technical, economic and allocative efficiency in peasant farming: evidence from the Dominican Republic". Developing economies. (1997)

Anang, B. et al. "Does farm size matter? Investigating scale efficiency of peasant rice farmers in northern Ghana". Economics Bulletin. (2016)

Goswami, B."Overcoming farm size induced constraints through endogenous institutional innovations: findings from a field study in Assam plains, India". Economics Bulletin. (2016)

Campos Ortiz, F.& Oviedo Pacheco, M."Extensión de los predios agrícolas y productividad. El caso del campo cañero en México". El trimestre económico. (2015)

Nwaru, J. C. et al. "Measurement and determinants of production efficiency among small-holder sweet potato (Ipomoea batatas) farmers in Imo State, Nigeria". European Journal of Scientific Research. (2011)

Bhattacharyya, A.& Mandal, R."A generalized stochastic production frontier analysis of technical efficiency of rice farming: A case study from Assam, India". Indian Growth and Development Review. (2016)

Goyal, S. K. et al. "An estimation of technical efficiency of paddy farmers in Haryana State of India". Indian Journal of Agricultural Economics. (2006)

Rao, C. A. R. et al. "Measuring and explaining technical efficiency in crop production in Andhra Pradesh". Indian Journal of Agricultural Economics. (2003)

Shanmugam, K. R. "Technical efficiency of rice growers in Bihar". Indian Journal of Applied Economics. (2000)

Codjoe, S. N. A."Population and food crop production in male and female-headed households in Ghana". International Journal of Development Issues. (2010)

Wang, C. et al. "Effective mechanism of non-agricultural differentiation of farmers occupation on arable land utilization efficiency". International Journal of Earth Sciences and Engineering. (2016)

Panda, s."Farmer education and household agricultural income in rural India". International Journal of Social Economics. (2015)

Adeyemo, R.& Akinola, A. A."Productivity of cassava, yam and maize under tropical conditions". International Journal of Vegetable Science. (2010)

Ajekiigbe, N. A. et al. "Technical efficiency in production of underutilized indigenous vegetables". International Journal of Vegetable Science. (2018)

Galawat, F.& Yabe, M."Evaluation of technical, allocative, and economic efficiency in rice production; a case study on rice farmers in Brunei Darussalam". Journal- Faculty of Agriculture Kyushu University. (2012)

Battese, G. E. et al. "Factors influencing the productivity and efficiency of wheat farmers in Punjab, Pakistan". Journal of agribusiness in developing and emerging economies. (2017)

Okuyama, Y. et al. "Technical efficiency and production potential of selected cereal crops in Senegal". Journal of agriculture and rural development in the tropics and subtropics. (2017)

Hakorimana, F.& Akcaoz, H."The functional analysis of maize production and the effect of land consolidation on the productivity in Rwanda". Journal of Animal & Plant Sciences. (2018)

Armagan, G.& Ozden, A."Determinations of total factor productivity with cobb-douglas production function in agriculture: the case of Aydin-Turkey". Journal of Applied Sciences. (2007)

Hadush, M. "Implication of animal feed and water scarcity on labor allocation, food production and per capita food consumption in Tigrai region, Ethiopia". Journal of Economic Development. (2017)

Alam, A. et al. "Stochastic frontier approach to measure technical efficiency of two irrigation systems in Gilgit district, Gilgit-Baltistan region of Pakistan". Journal of Food, Agriculture and Environment. (2012)

Amaza, P. S.& Ogundari, K."An investigation of factors that influence the technical efficiency of soybean production in the Guinea savannas of Nigeria". Journal of Food, Agriculture and Environment. (2008)

Baig, N. et al. "Impact of modern irrigation practices on farm productivity in arid regions of Pakistan". Journal of Food, Agriculture and Environment. (2014)

Nargis, F.& Lee, S. H."A non-parametric efficiency analysis of Boro and Aman rice production in selected areas of the Mymensingh district in Bangladesh". Journal of Food, Agriculture and Environment. (2013)

Hartoyo, S."The impact of rural road rehabilitation on rice productivity and farmers income in Kemang village, Cianjur, West Java, Indonesia". Journal of ISSAAS (International Society for Southeast Asian Agricultural Sciences). (2013)

Kundu, S."Inverse farm size-productivity relationship: A test using regional data across two time-points". Journal of Rural Development. (2011)

Alam, M. J. et al. "Tracing the impact of market reform on productivity growth of rice at the farm level in Bangladesh". Journal of the Asia Pacific Economy. (2014)

Anik, A. R.& Salam, M. A."Assessing and explaining vegetable growers´ efficiency in the south-eastern hilly districts of Bangladesh". Journal of the Asia Pacific Economy. (2017)

Mahadevan, R.& Gonemaituba, W."A note on soil erosion and the loss in crop production: case study of Fiji's ginger farms". Journal of the Asia Pacific Economy. (2013)

Mar, S. et al. "Technical efficiency analysis of mango production in Central Myanmar". Journal of the International Society for Southeast Asian Agricultural Sciences,. (2013)

Thiruchelvam, S."Efficiency of rice production and issues relating to cost of production in the districts of Anuradhapura and Polonnaruwa". Journal of the National Science Foundation of Sri Lanka. (2005)

Kane, G. Q. et al. "Efficiency of Groundnuts/Maize Intercropped Farms in Zoetele, South Cameroon: A Data Envelopement Approach". Life Science Journal. (2012)

Lekunze, J. et al. "Socio-economic constraints to sunflower production in Bojanala farming community of the North-West province, South Africa". Life Science Journal. (2011)

Esham, M."Technical efficiency and determinants of maize production by smallholder farmers in the Moneragala district of Sri Lanka". Mediterranean Journal of Social Sciences. (2014)

Sjakir, M. et al. "Learning and technology adoption impacts on farmer's productivity". Mediterranean Journal of Social Sciences. (2015)

Chemak, F.& Dhehibi, B."Efficacité technique des exploitations en irrigué. Una approche paramétrique versus non paramétrique". New Medit. (2010)

Chemak, F.& Dhehibi, B."Efficacité technique des exploitations en irrigué. Una approche paramétrique Versus non paramétrique". New Medit. (2010)

Ochieng, J. et al. "Effects of climate variability and change on agricultural production: the case of small scale farmers in Kenya". NJAS - Wageningen Journal of Life Sciences. (2016)

Tan, S. et al. "Impact of land fragmentation on rice producers' technical efficiency in South-East China". NJAS - Wageningen Journal of Life Sciences. (2010)

Kambhampati, U. et al. "Farm-level performance of genetically modified cotton. A frontier analysis of cotton production in Maharashtra". Outlook on Agriculture. (2006)

Qasim, M.& Knerr, B."Determinants of Farm Revenues from Rainfed Agriculture in the Punjab, Pakistan". Outlook on Agriculture. (2013)

Admassie, A."Sources of efficiency differentials in smallholder agriculture". Quarterly Journal of International Agriculture. (1999)

Ajibefun, I. A."Cropping system, technical efficiency and policy options: A stochastic frontier analysis of Nigerian small-scale farmers". Quarterly Journal of International Agriculture. (2006)

Ajibefun, I. A. et al. "Determinants of technical efficiency in smallholder food crop farming: application of stochastic frontier production function". Quarterly Journal of International Agriculture. (2002)

Ferenji, B. T.& Heidhues, F."Fall in technical efficiency of small farm households in the post reform period". Quarterly Journal of International Agriculture. (2007)

Alene, A. D. et al. "The technical and cost efficiencies of hybrid maize production in western Ethiopia". Quaterly Journal of International Agriculture. (2005)

Alene, A. D. et al. "The extent and determinants of production efficiency of farmers in the rainforest margins in Central Sulawesi, Indonesia: implications for land use and support services". Quaterly Journal of International Agriculture. (2005)

Bangwayo-Skeete, P. F. et al. "Are Zimbawe's fast track land reform farms more technically efficient than communal farms?". Quaterly Journal of International Agriculture. (2010)

Nguezet, P. M. D. et al. "Impact of improved rice technology (NERICA varieties) on income and poverty among rice farming households in Nigeria: a local average treatment effect (LATE) approach". Quaterly Journal of International Agriculture. (2011)

Dasgupta, S. et al. "Is environmentally friendly agriculture less profitable for farmers? Evidence on integrated pest management in Bangladesh". Review of Agricultural Economics. (2007)

Aye, G. C.& Mungatana, E. D."Evaluating the performance of small scale maize producers in Nigeria: an integrated distance function approach". Review of Urban & Regional Development Studies. (2013)

Marques de Magalhaes, M. et al. "Land reform in NE Brazil: a stochastic frontier production efficiency evaluation". Revista de Economia e Sociologia Rural. (2011)

Trujillo, J. C.& Iglesias, W. J."Measurement of the technical efficiency of small pineapple farmers in Santander, Colombia: a stochastic frontier approach". Revista de Economia e Sociologia Rural. (2013)

Audibert, M. et al. "Effets économiques du paludisme sur les cultures de rente: l'exemple du café et du cacao en Côte d'Ivoire". Revue d'économie du développement. (2009)

Combary, O. S.& Savadogo, K."Les sources de croissance de la productivité globale des facteurs dans les exploitations cotonnières du Burkina Faso". Revue d'économie du développement. (2014)

Gebrehiwot, K. G."The impact of agricultural extension on farmers' technical inefficiencies in Ethiopia: a stochastic production frontier approach". South African Journal of Economic and Management Sciences. (2017)

Pradhan, K. C.& Mukherjee, S."Examining technical efficiency in Indian agricultural production using production frontier model". South Asia Economic Journal. (2018)

Mahmood, H. Z. et al. "Re-examining the inverse relationship between farm size and productivity in Pakistan". The Journal of Animal and Plant Sciences. (2014)

Ahmad, M. et al. "Wheat productivity, efficiency and sustainability: a stochastic production frontier analysis". The Pakistan Development Review. (2002)

Anriquez, G.& Valdés, A."Determinants of Farm Revenue in Pakistan". The Pakistan Development Review. (2006)

Without impact factor

Chepng'etich, E. et al. "Factors that influence technical efficiency of sorghum production: a case of small holder sorghum producers in lower eastern Kenya". Advances in Agriculture. (2015)

Kurukulasuriya, P.& Mendelsohn, R."A Ricardian analysis of the impact of climate change on Africa cropland". African Journal of Agricultural and Resource Economics. (2008)

Liverpool-Tasie, L. S. O. et al. "Productivity effects of sustainable intensification: The case of Urea deep placement for rice production in Niger State, Nigeria". African Journal of Agricultural and Resource Economics. (2015)

Tchale, H." The efficiency of smallholder agriculture in Malawi". African Journal of Agricultural and Resource Economics. (2009)

Ogundari, K.& Brümmer, B."Estimating Technical Efficiency, Input substitution and complementary effects using Output Distance Function: A study of Cassava production in Nigeria". Agricultural Economics Review. (2011)

Croppenstedt, A.& Demeke, M."An empirical study of cereal crop production and technical efficiency of private farmers in Ethiopia: a mixed fixed-random coefficients approach". Applied Economics. (1997)

Scandizzo, P. L.& Savastano, S."Revisiting the farm size-productivity relationship: new evidence from sub-Saharan countries.". BOOK: Agriculture and Rural Development in a Globalizing World: Challenges and Opportunities. (2017)

Nakano, Y. et al. "The possibility of a rice green revolution in large-scale irrigation schemes in Sub-Saharan Africa". Book: An African Green Revolution: Finding ways to boost productivity on small farms. (2013)

Tchamou Meughoyi, C."Improved Seeds and Agricultural Productivity of Family Farms in Cameroon". Book: Building a Resilient and Sustainable Agriculture in Sub-Saharan Africa. (2018)

Suyanto, S. et al. "Agroforestry management in Sumatra". Book: land tenure and natural resource management. (2001)

Quisumbing, A. et al. "Agroforestry management in Ghana". Book: Land tenure and natural resource management: a comparative study of agrarian communities in Asia and Africa. (2001)

Tatwangire, A.& Holden, S. T."Land tenure reforms, land market participation and the farm size- productivity relationship in Uganda". Book: Land Tenure reform in Asia and Afria. (2013)

Bachewe, F. N. et al. "Productivity and efficiency in high-potential areas". book: the economics of teff: exploring Ethiopia's biggest cash crop. (2018)

Udry, C."Efficiency and market structure: testing for profit maximization in African agriculture". Book: Trade, Growth and development. (1999)

Nkendah, R.& Temple, L."Pression démographique et efficacité technique des producteurs de banane plantain de l'Ouest-Cameroun". Cahiers Agriculture. (2003)

Ouedraogo, M. et al. "Economic impact assessment of climate change on agriculture in Burkina Faso: A Ricardian approach". Centre for Environmental Economics and Policy in Africa (University of Pretoria) - CEEPA Discussion paper. (2006)

Kurukulasuriya, P. et al. "Adaptation and Climate Change Impacts: A Structural Ricardian Model of Irrigation and Farm Income in Africa". Climate Change Economics. (2011)

Kassie, G. W."Agroforestry and land productivity: evidence from rural Ethiopia". Cogent Food & Agriculture. (2016)

Bevis, L. E.& Barrett, C. B."Close to the Edge: Do Behavioral Explanations Account for the Inverse Productivity Relationship?". Cornell University working paper. (2016)

Chattopadhyay, M.& Sengupta, A."Farm size and productivity: A new look at the old debate". Economic and Political Weekly. (1997)

Toufique, K. A."Farm size and productivity in Bangladesh agriculture. Role of transaction costs in rural labour markets". Economic and Political Weekly. (2005)

Fontan, C."Production et efficience technique des riziculteurs de Guinée. Une estimation paramétrique stochastique.". Économie rurale. Agricultures, alimentations, territoires. (2008)

Ngom, C. A. B. et al. "Mesure de l'efficacité technique des riziculteurs du bassin du fleuve Sénégal". Économie rurale. Agricultures, alimentations, territoires. (2016)

Nuama, E."L'efficacité technique des riziculteurs ivoiriens: la vulgarisation en question". Économie rurale. Agricultures, alimentations, territoires. (2010)

Arslan, A. et al. "Smallholder productivity under climatic variability: Adoption and impact of widely promoted agricultural practices in Tanzania". ESA Working paper. (2016)

Asfaw, S. et al. "Climate variability, adaptation strategies and food security in Malawi". ESA Working paper. (2014)

Croppenstedt, A."Measuring technical efficiency of wheat farmers in Egypt". ESA Working paper. (2005)

Karfadis, P. et al. "Effects of global warming on vulnerability to food insecurity in rural Nicaragua". ESA Working paper. (2011)

Karfakis, P. et al. "On the costs of being small: case evidence from Kenyan family farms". FAO Agricultural Development Economics Working Paper. (2017)

Singbo, A. G.& Lansink, A. O."Lowland farming system inefficiency in Benin (West Africa): directional distance function and truncated bootstrap approach". Food Security. (2010)

Abay, K. A. et al. "Correlated non-classical measurement errors, second best policy inference and the inverse size-productivity relationship in agriculture". IFPRI Discussion Paper. (2018)

Bowser, W.& Nelson, C. H."Land institutions, investments and income diversification". IFPRI Discussion Paper. (2012)

Deininger, K. et al. "Can labor market imperfections explain changes in the inverse farm size-productivity relationship?: Longitudinal evidence from rural India". IFPRI Discussion Paper. (2016)

Kumar, A. et al. "Impact of contract farming on profits of smallholders. Evidence from cultivation of onion, okra and pomegranate in India". IFPRI Discussion Paper. (2018)

Liu, Y. et al. "Structural transformation and intertemporal evolution of real wages, machine use, and farm size–productivity relationships in Vietnam". IFPRI Discussion Paper. (2016)

McCarthy, N.& Essam, T."Impact of water user associations on agricultural productivity in Chile". IFPRI Discussion Paper. (2009)

Nkonya, E. et al. "Policy options for increasing crop productivity and reducing soil nutrient depletion and poverty in Uganda". IFPRI Discussion paper. (2005)

Pender, J. et al. "Impacts of the Hutan Kamasyarakatan Social Forestry Program in the Sumberjaya Watershed, West Lampung district of Sumatra, Indonesia". IFPRI Discussion Paper. (2008)

Obunde, P. O. et al. "Effects of land tenure on agricultural productivity, and the environment. A case study of Suba and Laikipia districts". IFPRI Eastern Africa Food Policy Network. (2004)

Bagamba, F. et al. "Determinants of banana productivity and technical efficiency in Uganda". IFPRI Research Report. (2007)

Jansen, H. G. P. et al. "Rural development policies and sustainable use in the hillside areas of Honduras. A quantitative livelihoods approach". IFPRI Research Report. (2006)

Nisrane, F. et al. "Sources of inefficiency and growth in agricultural output in subsistence agriculture: a stochastic frontier analysis". IFPRI working paper. (2011)

Kraybill, D. et al. "Production and marketed surplus of crops in Uganda 1999-2006". IFPRI: Uganda Strategy Support Program Working Papers. (2012)

Helfand, S. M. et al. "Brazil's agricultural total factor productivity growth by farm size". Inter-American Development Bank Working Paper Series. (2015)

Ajibefun, I. A. et al. " Technical efficiency of small scale farmers: An application of the stochastic frontier production function to rural and urban farmers in Ondo State, Nigeria". International Economic Journal. (2006)

Bhatt, M. S.& Bhat, S. A."Technical efficiency and farm size productivity- micro level evidence from Jammu and Kashmir". International Journal of Food and Agricultural Economics. (2014)

Agada, G. O. et al. "Effect of microfinance Bank's credit on cereal crops productivity in Federal capital territory, Abuja". International Journal of Scientific and Technology Research. (2018)

Naceur, M.& Mongi, S."The technical efficiency of collective irrigation schemes in south-eastern of Tunisia". International Journal of Sustainable Development & World Policy. (2013)

Gadedjisso-Tossou, A. et al. "Assessing the impact of climate change on smallholder farmers' crop net revenue in Togo". Journal of Agriculture and Environment for International Development. (2016)

Addai, K. N.& Owusu, V."Technical efficiency of maize farmers across various agro ecological zones of ghana". Journal of Agriculture and Environmental Sciences. (2014)

Biam, C et al. "Economic efficiency of small scale soyabean farmers in Central Agricultural Zone, Nigeria: A Cobb-Douglas stochastic frontier cost function approach". Journal of Development and Agricultural Economics. (2016)

Adeoti, A. I. "Farmers' efficiency under irrigated and rainfed production systems in the derived savannah zone of Nigeria". Journal of Food, Agriculture and Environment. (2006)

Boubacar, O. et al. "Analysis on Technical Efficiency of Rice Farms and its influencing factors in South-Western of Niger". Journal of Norheast Agricultural University. (2016)

Ashfaq, M. et al. "Contribution of rural women in the farm productivity". Journal of Plant and Animal Sciences. (2008)

Mahesh, R."Farm size- productivity relationship: some evidence from Kerala". Kerala Institute for Environment and Development Working Paper. (2000)

DeSilva, S."Access to markets and farm efficiency: a study of rice farms in the bicol region, philippines". Levy Economics Institute of Bard College Working Paper. (2011)

Masterson, T."Productivity, technical efficiency, and farm size in Paraguayan agriculture". Levy Economics Institute of Bard College Working Paper. (2007)

Ünal, F. G. "Small is Beautiful: Evidence of an Inverse Relationship Between Farm Size and Yield in Turkey". Levy Economics Institute of Bard College Working Paper. (2008)

Thapa, S."The relationship between farm size and productivity: empirical evidence from the Nepalese mid-hills". MPRA paper. (2007)

Kamara, A. B. et al. "Economic viability of small-scale irrigation systems in the context of state withdrawal: the Arabie scheme in the Northern province of South Africa". Physics and Chemistry of the Earth. (2002)

Khan, H.& Shah, M."Irrigation, farm productivity and poverty reduction in KPK: understanding direct and indirect impacts and linkages". Procedia Economics and Finance. (2012)

Abate, G. T. et al. "The impact of the use of new technologies on farmers wheat yield in Ethiopia: evidence from randomized controlled trial". Research for Ethiopia's agriculture policy: analytical support for the agricultural transformation agency. (2014)

Ojiako, I. A. et al. "Determinants of productivity of smallholder farmers supplying cassava to starch processors in Nigeria: a baseline evidence". Russian Journal of Agriculture and Socioeconomic Sciences. (2017)

Tariq, M. et al. "An estimation of technical efficiency of strawberry production in district Charsadda Khyber Pakhtunkhwa". Sarhad Journal of Agriculture. (2018)

Ud-din, R.& Khattak, N. U. R."Impacts of farm mechanisation on wheat and maize crops' productivity in Peshawar valley". Sarhad Journal of Agriculture. (2018)

Aneani, F.& Ofori-Frimpong, K."An analysis of yield gap and some factors of cocoa (Theobroma cacao) yields in Ghana". Sustainable Agriculture Research. (2013)

Romanillos, R. D. et al. "Community development strategies and other factors affecting rice productivity in inland valleys in Quezon province, Luzon, Philippines". The Asian International Journal of Life Sciences. (2016)

Gragasin, M. et al. "Irrigators' Association and Farm Productivity: A Comparative Study of Two Philippne Irrigation Systems". The Japanese Journal of Rural Economics. (2005)

Burki, A. A.& Shah, H. N."Stochastic Frontier and Technical Efficiency of farms in irrigated areas of Pakistan's Punjab". The Pakistan Development Review. (1998)

Foster, A. D.& Rosenzweig, M. R."Are Indian farms too small? Mechanization, agency costs, and farm efficiency". Unpublished Manuscript, Brown University and Yale University. (2011)

Alkahtani, S. H.& Elhendy, A. M."Organic and conventional date farm efficiency estimation, and its determents at Riyadh province, Kingdom of Saudi Arabia". WIT Transactions on Ecology and the environment. (2012)

Jacoby, H.& Minten, B."Land titles, investment and agricultural productivity in Madagascar". World Bank Economic and Sector Work Studies. (2006)

White, W. et al. "An impact evaluation of India's second and third Andhra Pradesh irrigation projects". World Bank Independent Evaluation Group. (2008)

Ali, D. A. et al. " Costs and benefits of land fragmentation: evidence from Rwanda". World Bank Policy Research Working Paper. (2015)

Ali, D. A. et al. "Credit constraints, agricultural productivity, and rural nonfarm participation. Evidence from Rwanda". World Bank Policy Research Working Paper. (2014)

Backiny-Yetna, P.& McGee, K."Gender differentials and agricultural productivity in Niger". World Bank Policy Research Working Paper. (2015)

Brambilla, I. & Porto, G. G."Farm productivity and market structure: Evidence from cotton reforms in Zambia". World Bank Policy Research Working Paper. (2006)

Cadot, O. et al. "How costly is it for poor farmers to lift themselves out of subsistence?". World Bank Policy Research Working Paper. (2006)

Deininger, K. et al. "Smallholders' land ownership and access in sub-Saharan Africa. A new landscape?". World Bank Policy Research Working Paper. (2015)

Deininger, K. et al. "Market and nonmarket transfers of land in Ethiopia. Implications for efficiency, equity, and nonfarm development". World Bank Policy Research Working Paper. (2003)

Desiere, S& Jolliffe, D."Land productivity and plot size: Is measurement error driving the inverse relationship?". World Bank Policy Research Working Paper. (2018)

Dillon, A. et al. "Land measurement bias and its empirical implications. Evidence from a validation exercise". World Bank Policy Research Working Paper. (2016)

Do, Q.& Iyer, L."Land rights and economic development. Evidence from Vietnam". World Bank Policy Research Working Paper. (2003)

Eid, H. M. et al. "Assessing the economic impacts of climate change on agriculture in Egypt. A Ricardian approach". World Bank Policy Research Working Paper. (2007)

Gomez Osorio, C. et al. "Who is benefitting from fertilizer subsidies in Indonesia?". World Bank Policy Research Working Paper. (2011)

Jain, S."An empirical economic assessment of impacts of climate change on agriculture in Zambia". World Bank Policy Research Working Paper. (2007)

Larson, D. F. et al. "Are women less productive farmers? How markets and firms affect fertilizer use, productivity and measured gender effects in Uganda". World Bank Policy Research Working Paper. (2015)

Mano, R.& Nhemachena, C."Assessment of the economic impacts of climate change on agriculture in Zimbabwe: A Ricardian approach". World Bank Policy Research Working Paper. (2007)

Oseni, G. et al. "Can agricultural household farm their way out of poverty?". World Bank Policy Research Working Paper. (2014)

Savastano, S.& Scandizzo, P. L."Farm size and productivity: A direct-inverse-direct relationship". World Bank Policy Research Working Paper. (2017)

Soloaga, I"The treatment of non-essential inputs in a cobb-douglas technology. An application to Mexican rural household-level data". World Bank Policy Research Working Paper. (2000)

Verner, D."Labor markets and income generation in rural Argentina". World Bank Policy Research Working Paper. (2006)

S6: Including total values or total output

Indicators of farm performance are not a homogeneous group: Gross output (total production, total revenue, yield, value per area), net value (ratio revenues/cost, total profit, gross margin, net farm income, profit per area, gross margin per area, net farm income per area) and efficiency.

Total production or total revenue indicators can be included and interpreted alongside per area indicators (i.e. yield or value per area), depending on the information provided and the functional form of the relationship between the performance indicator and the land size. For technical details, please refer to the Box below.

Box S4. Interpreting total values or output

When assessing the coefficient (β) related to crop area in relation to indicators per area (e.g. yield), the interpretation as to the association between the size of a farm or plot and its performance is intuitive: when β is lower than zero an IR appears, whereas if β is greater than zero, a direct relationship emerges. When dealing with total values, interpretation is less straightforward. This is particularly the case when indicator is total output **(y)** and area is **(x)**, as in the following example:

$$\boldsymbol{y = ax^b}$$
$$\boldsymbol{\ln y = \ln a + b \ln x}$$
$$\boldsymbol{\ln y - \ln x = \ln a + b \ln x - \ln x}$$
$$\boldsymbol{\ln \frac{y}{x} = a' + (b-1) \ln x}$$
$$\boldsymbol{\ln y = \ln a + \beta \ln x} \quad \text{where} \quad \boldsymbol{\beta = b - 1}$$

If **b=0, then β=-1**. If the area (x) increases, the production (y) does not vary, so the yield is being reduced, hence decreasing marginal productivity and an extreme version of inverse relationship.
If **0<b<1, then -1<β<0**. If the area (x) increases, the production (y) varies in lower proportion, so the yield is being reduced, hence inverse relationship
If **b=1, then β=0**. If the area (x) increases, the production (y) varies in the same proportion, so the yield does not vary.
If **b>1, then β>0**. If the area (x) increases, the production (y) increases in greater proportion, so the yield is being increased, hence a direct relationship.

S7: Single performance indicators and aggregated group

Table S 2. Indicators to assess agricultural performance

Indicator	Group
Total production*	***Gross Output***
Total revenue*	
Yield (production per area)	
Value per area	
Ratio revenues/cost	***Net value***
Total profit	
Gross margin	
Net farm income	
Profit per area	
Gross margin per area	
Net farm income per area	
Efficiency (technical, economic, etc.)	***Efficiency***
Total factor productivity (TFP)	

*For technical details as to how the estimates of these indicators are used, please refer to S6: Including total values or total output

Note as an example that in the category of 'gross output' we have the following econometric relationship: Yields = $\beta_0 + \beta_1 \cdot$ land size + other control variables. In this case, crop yield is regressed on farm size, considering a relationship between agricultural output and land size. By contrast, in the category of 'net value' we have the following econometric relationship: Profit per area = $\beta_0 + \beta_1 \cdot$ land size + other control variables. As profit includes both revenues (hence output) and costs, land size influences both. The 'efficiency' category is comparable to the 'net value' approach, since land size is assumed to have an effect on both output and input use.

S8: Definition of variables

Table S 3. Definition of variables for meta-regression

Variable	Definition	Type of variable
Farm level	Studies where the performance refers to a farm as a whole, including all exploited land.	Dummy
Plot level	Studies where the performance refers to a single plot among all plots of a farm. Generally, this refers to the main plot of a given farm.	Dummy
Indicator group (ref: *Gross output*)	Category of study grouped by type indicators gathering those associated with gross measures of performance with either physical or monetary units.	Categorical
Net value	Category of study grouped by type indicators gathering those associated with monetary values of performance, net of costs (i.e. inputs, labor, etc.), variable and/or fixed.	
Efficiency and TFP analyses	Category of study grouped by type indicators gathering those associated with ranking exercises of the relative efficiency of farms or plots, including Total Factor Productivity (TFP) studies.	
Data source (ref: *LSMS*)	Living Standards Measurement Study-Integrated Surveys on Agriculture (LSMS-ISA), nation-wide population-based farm survey dataset supported by the World Bank. / ***This variable is also a proxy for the size range of farms.***	Categorical
National Statistics	Data from regular surveys, census data not related to LSMS.	
Ad-hoc surveys	Ad hoc surveys generally developed by research organizations.	

Variable	Definition	Type of variable
Warm arid and semi-arid tropics	The study was located in an area characterized as warm arid agro-ecological zone (AEZ). The remaining areas or mixed areas are categorized as (0).	Dummy
Time	This variable captures the "age" of a given dataset with reference to 2017, the most recent year. Time = (2017-last year of dataset). Please note that a study may refer to a single year, a pooled sample or a panel data over several years. The most recent year is considered in the record.	Ordinal variable
Subsistence farming (ref: *Very small, Average [0,1] ha*)	Variable dividing the studies by the average land size of the study (farm size). ***It is also a proxy for the size range of farms.*** / This category gathers the studies with sizes averaging 1 hectare or less.	Categorical
Subsistence farming (Small, Average (1,2] ha	This category gathers the studies with sizes averaging between (1 - 2] hectares. It has not to be mixed with land size of each farm or plot.	
Average >2 ha	This category gathers the studies with sizes averaging more than 2 hectares. It has not to be mixed with land size of each farm or plot.	
Main crop (ref: *Maize*)	Indicates whether the study has maize as the main crop of interest.	Categorical
Rice	Indicates whether the study has rice as the main crop of interest.	
Wheat	Indicates whether the study has wheat as the main crop of interest.	
Other crops	Indicates whether the study has other or undefined crops of interest.	
**Variable**	**Definition**	**Type of variable**

Specialized paper	Indicates if the study is extracted from a paper dedicated to the exploration and discussion of the land area-performance relationship (1) or if it originates from a paper exploring farm or plot performance but without special emphasis on land size-performance relationship (0).	Dummy
GPS measurement	Indicates if the land area is measured by GPS (1) or relies on farmer's estimates (0).	Dummy
Inclusion of soil quality and/or slope	Indicates whether the specification of the model accounts for soil quality and/or the slope of the land (1 if accounted for, 0 if not).	Dummy
Inclusion of irrigation	Indicates whether the specification of the model accounts for whether farmers use irrigation (1 if accounted for, 0 if not).	Dummy
Inclusion of mechanical and/or animal power	Indicates whether the specification of the model accounts for the physical capital of farmers, there be mechanical or animal power (1 if accounted for, 0 if not).	Dummy
Inclusion of access to credit	Indicates whether the specification of the model accounts for whether farmers access financial services (credit and insurance services) (1 if accounted for, 0 if not).	Dummy
Inclusion of off-farm income/ activity	Indicates whether the specification of the model accounts for off-farm activities, opportunities or income. (1 if accounted for, 0 if not)	Dummy
Panel	Indicates whether the specification of the model was developed with panel data explicitly implementing methods for controlling time-invariant unobservable characteristics (e.g. fixed effects). (1 if panel, 0 if not)	Dummy

S9: Clustering standard errors: choosing the cluster level

The nature of the evidence used in the analysis requires controlling for the lack of independence of standard errors because cases may share time-invariant characteristics within clusters if collected in the same country or even region (SSA, Asia, MENA or LAC).

To test the relevance of a given level of control (country vs region), we estimated Interclass Correlation Coefficient (ICC) using a multilevel logistic regression (melogit). ICC is estimated as post-estimation from a simple unconditional mean model with only the dependent variable, (i.e. IR or not) without any independent variable.

ICC is between 0 and 1. ICC=1 would indicate that all differences are explained by between-cluster differences (e.g. countries), whereas negligible or non-significant estimate would not warrant taking clustering into account.

It is also possible to use the approach proposed by Muthén and Satorra (*95*) by calculating the design effect with the formula (1+(average group size-1)*ICC. A design effect > 2 is suggesting that clustering should not be ignored (*96*).

Table S 4. Tests to choose the cluster level

	Test, IR - Direct			Test, IR - all others		
	**ICC**	**Average group size**	**Design effect**	**ICC**	**Average group size**	**Design effect**
Region	0.0022353	151.5	1.336413	0.0050501	199	1.99992
Country	0.2443101***	10.6	3.345377	0.184986***	13.3	3.275483
N	*606*	*606*	*606*	*796*	*796*	*796*

Accounting for the two approaches, we retain clustering by country as the main strategy for our study as its ICC is systematically significant and above 2, following the Design effect approach. The estimates for region are less clear and robust between the two data sub-sets.

S10: Weighting strategy

To control on the quality of how the aggregated results are obtained, a weighting strategy was introduced alongside the unweighted model. The unweighted model gives the same importance to all cases whereas a weighting strategy gives full weight to those cases developed with the highest levels of the chosen criteria and less influence to those that are less performant in the given criteria.

We used the general quality criteria of the source of the publication (between peer- and non-reviewed and among peer-reviewed publications) as the criteria for allocating weights. Studies from the most reputable publications are given more importance than those with less academic prestige or without being submitted to a peer-review process.

Quality of publication criteria

Here the emphasis is put on the methodological and general quality of the publication of the study. According to this criterion, three categories of publications were distinguished. The first group, provinding full points (weight equals to 1), gathers academic peer-reviewed studies published in the most reputable publications within the 1st and 2nd quartile of their respective categories, according to Scimago Journal Rank (SJR). The weight is divided by half for the 3rd and 4th quartile categories. Finally, the publications without impact factor or not being systematically peer-reviewed, are given ¼ of the points. This last group includes working papers from established instutions in the field (e.g. IFPRI, World Bank, etc. as decribed in the selection and sources section) along relevant technical reports and books.

S11 Models Farm-level:

Table S 5. Stata output: Farm level models, Logit estimates and predicted marginal probabilities

Refer to Excel file “Models.xlsx”, Farm level models: Sheet: “Farm level”, as Other Supplementary material, for a handier view.

Model	1	2	3	4	5	6	7	8	9	10	11	12	13	14	15	16	17	18	19	20	21	22	23	24	25	26	27
Dependent variable (Dummy, 0;1): 1:Recording Inverse relationship; 0:Direct relationship estimate.	x	x			x				x		x	x		x		x			x		x	x		x		x	
Dependent variable (Dummy, 0;1): 1:Recording Inverse relationship; 0: All other recorded estimates, including non-significant ones			x	x		x	x	x		x			x		x		x	x		x			x		x		x
Full model (all variables)											x	x	x	x	x	x	x					x	x	x	x	x	x
Sample limited to cases with information on subsistence status		x		x			x	x			x	x	x	x	x	x	x					x	x	x	x	x	x
VCE cluster (country)					x	x	x	x	x	x	x	x	x	x	x	x	x	x	x	x	x	x	x	x	x	x	x
Weighting (quality of publication)								x	x	x		x	x			x	x			x	x	x	x	x	x	x	x
Grey literature included									x	x				x	x	x	x		x		x			x	x	x	x
Main model																x	x							x	x	x	x
Indicator group (ref: Gross output)																											
Net value	-0.462	-0.631*	-0.644**	-0.929***	-0.462	-0.631	-0.929**	-0.872**	-0.612	-0.643*	-0.734+	-0.781+	-1.240***	-0.814*	-1.223***	-0.804+	-1.227***	-0.805*	-0.771**	-0.622+	-0.657*	-0.792+	-1.240***	-0.811+	-1.228***	-0.816*	-1.250***
Efficiency	-1.490***	-1.546***	-1.448***	-1.681***	-1.490***	-1.546***	-1.681***	-1.802***	-1.657***	-1.551***	-1.610***	-1.717***	-1.936***	-1.765***	-1.886***	-1.768***	-1.947***	-1.655***	-1.537***	-1.653***	-1.539***	-1.701***	-1.939***	-1.755***	-1.948***	-1.631***	-1.828***
Data source (ref: LSMS)																											
National Statistics											-1.677*	-1.351+	-1.190**	-1.336*	-1.306**	-1.252+	-1.157**					-1.423+	-1.186**	-1.317+	-1.156**	-1.184	-1.104*
Ad-hoc surveys											-2.301***	-2.295***	-1.963***	-2.021***	-1.896***	-2.207***	-1.945***					-2.331***	-1.961***	-2.240***	-1.944***	-2.085***	-1.831***
Warm arid and semi-arid tropics											-0.119	-0.193	-0.302	-0.090	-0.161	-0.185	-0.274					-0.191	-0.303	-0.183	-0.274	-0.234	-0.317
Last year of study											-0.051**	-0.055**	-0.062***	-0.052**	-0.062***	-0.055**	-0.064***					-0.055**	-0.062***	-0.055**	-0.064***	-0.055**	-0.063***
Panel, time invariant controls																						0.464	-0.033	0.405	-0.016		
Subsistence farming (ref: Very small, Average [0;1]] Ha.)																											
Subsistence farming (Small, Average]1;2] Ha.											-0.020	-0.280	-0.244	0.108	-0.139	-0.233	-0.260					-0.273	-0.243	-0.225	-0.26	-0.295	-0.324
Average >2 Ha.											-0.436	-0.306	-0.405	-0.321	-0.409	-0.271	-0.397					-0.304	-0.405	-0.267	-0.397	-0.44	-0.506+
Main crop (ref: Maize)																											
Other crops											0.876*	0.729	0.438	0.887*	0.644*	0.735+	0.445					0.719	0.44	0.726	0.446	0.736	0.444
Rice											1.225*	1.096*	0.463	1.215*	0.631	1.094*	0.466					1.082*	0.464	1.082*	0.467	0.998+	0.408
Wheat											0.507	0.287	-0.198	0.458	-0.195	0.278	-0.256					0.296	-0.198	0.287	-0.256	0.155	-0.338
Specialised paper											-0.596	-0.626	-0.263	-0.572	-0.434	-0.624	-0.312	0.033	0.024	0.085	0.222	-0.679	-0.26	-0.669	-0.31	-0.649	-0.302
GPS measurement											0.785	1.141	0.953	1.297	1.067	1.335	1.125					1.197	0.949	1.355	1.124	1.211	1.048
Inclusion of soil quality and/or slope											-0.404	-0.405	-0.492	-0.293	-0.490	-0.372	-0.505					-0.369	-0.494	-0.34	-0.506	-0.329	-0.46
Inclusion of irrigation											0.057	0.040	-0.201	-0.039	-0.119	0.009	-0.192					0.058	-0.203	0.023	-0.193	-0.059	-0.237
Inclusion of mechanical and/or animal power											-0.074	-0.052	-0.381	-0.008	-0.212	-0.024	-0.314					-0.072	-0.379	-0.037	-0.313	-0.122	-0.37
Inclusion of access to credit											-0.154	-0.042	-0.127	-0.104	-0.183	-0.020	-0.108					-0.021	-0.129	-0.001	-0.109	-0.033	-0.071
Inclusion of off-farm activities/income																										-0.926	-0.777
constant	1.040***	1.113***	0.388***	0.563***	1.040***	1.113***	0.563**	0.605**	1.161***	0.441*	2.239*	2.307*	1.835**	1.917*	1.591**	2.200*	1.792**	1.158***	0.451*	1.145***	0.398+	2.310*	1.835**	2.203*	1.792**	2.343**	1.865***
Log p-likelihood	-310.849	-256.779	-443.929	-357.521	-310.849	-256.779	-357.521	-317.105	-268.174	-392.867	-234.266	-194.699	-275.483	-256.268	-366.711	-200.149	-284.913	-355.809	-515.190	-268.121	-392.342	-194.234	-275.479	-199.774	-284.912	-197.133	-282.101
pseudo-R^2	0.066	0.069	0.062	0.085	0.066	0.069	0.085	0.090	0.077	0.066	0.151	0.166	0.176	0.168	0.181	0.171	0.182	0.078	0.066	0.077	0.067	0.168	0.176	0.173	0.182	0.184	0.19
N	514	431	683	564	514	431	564	648	606	796	431	431	564	498	648	498	648	606	796	606	769	431	564	498	648	498	648
AIC	627.699	519.559	893.857	721.042	627.699	519.559	721.042	640.210	542.347	791.734	504.532	425.397	586.966	548.536	769.423	436.298	605.826	719.619	1038.379	544.242	792.684	426.468	588.958	437.548	607.825	432.267	602.201
BIC	640.425	531.757	907.437	734.047	640.425	531.757	734.047	653.632	555.568	805.772	577.722	498.587	664.997	624.327	849.953	512.089	686.356	737.246	1057.098	561.869	811.402	503.724	671.324	517.549	692.828	512.268	687.205

S12: Models plot-level:

Table S 6. Stata output: Plot level models, Logit estimates and predicted marginal probabilities

Refer to Excel file "Models.xlsx", Plot level models: Sheet: "Plot level", as Other Supplementary material, for a handier view.

**Model**	**1**	**2**	**3**	**4**	**5**	**6**	**7**	**8**	**9**	**10**	**11**	**12**	**13**	**14**	**15**	**16**
Dependent variable (Dummy, 0;1): 1:Recording Inverse relationship; 0:Direct relationship estimate.	x		x			x	x		x		x			x		x
Dependent variable (Dummy, 0;1): 1:Recording Inverse relationship; 0: All other recorded estimates, including non-significant ones		x		x	x			x		x		x	x		x	
Full model (all variables)						x	x	x	x	x	x	x				
Sample limited to cases with information on subsistence status																
VCE cluster (country)			x	x	x	x	x	x	x	x	x	x	x	x	x	x
Weighting (quality of publication)					x		x	x			x	x			x	x
Grey literature included									x	x	x	x		x		x
Main model									x	x	x	x				
Indicator group (ref: Gross output)																
Net value	0.384	-0.428	0.384	-0.428	-0.584	-0.614	-0.857	-1.226	-1.134	-1.580**	-1.057	-1.406*	-0.408	-0.942+	-0.174	-0.944+
Efficiency	-2.073***	-1.814***	-2.073***	-1.814***	-2.062***	-1.883*	-2.197*	-2.203*	-2.225***	-2.170**	-2.293**	-2.260**	-2.222***	-1.957***	-2.385***	-2.070***
Data source (ref: LSMS)																
National Statistics						0.083	0.259	-0.157	-0.097	0.040	0.176	-0.004				
Ad-hoc surveys						-0.817	-0.521	-0.208	-0.217	0.110	-0.286	0.009				
Warm arid and semi-arid tropics						1.638*	1.739*	0.474	0.924	0.396	1.362+	0.388				
Last year of study						-0.063	-0.075	-0.076*	-0.072	-0.080**	-0.076	-0.076**				
Main crop (ref: *Maize*)																
Other crops						1.933**	1.716*	1.267**	1.398*	1.229*	1.508*	1.201**				
Rice						0.734	0.297	-0.282	0.513	-0.028	0.217	-0.312				
Wheat						0.736	0.551	-0.395	1.012	0.269	0.635	-0.271				
Specialised paper						1.388*	1.747**	1.251*	0.956+	1.187**	1.551**	1.323**	0.510	0.838**	1.049+	1.187***
GPS measurement						-1.275	-1.230	-0.873	-1.166	-0.879	-1.213	-0.871				
Inclusion of soil quality and/or slope						-0.794	-0.653	-0.923+	-0.349	-0.689	-0.510	-0.807+				
Inclusion of irrigation						-0.487	-0.562	0.132	-0.281	0.123	-0.435	0.140				
Inclusion of mechanical and/or animal power						-0.586	-0.784	0.555	-0.484	0.449	-0.703	0.498				
Inclusion of access to credit						-0.453	-0.514	-0.136	-0.066	0.047	-0.361	-0.077				
Constant	1.156***	0.428*	1.156***	0.428	0.491	0.506	0.387	-0.343	0.127	-0.755	0.238	-0.519	1.190***	0.398	1.112***	0.293
Log p-likelihood	-84.870	-133.019	-84.870	-133.019	-123.479	-69.604	-58.513	-96.337	-90.252	-137.177	-64.271	-103.222	-100.751	-155.145	-74.055	-119.088
pseudo-R²	0.100	0.061	0.100	0.061	0.067	0.262	0.284	0.231	0.198	0.192	0.255	0.220	0.104	0.086	0.142	0.100
N	155	205	155	205	247	155	155	205	190	247	190	247	190	247	190	247
AIC	175.739	272.039	175.739	272.039	252.957	171.209	149.025	224.674	212.504	306.355	160.542	238.443	209.501	318.291	156.111	246.176
BIC	184.870	282.008	184.870	282.008	263.486	219.903	197.720	277.842	264.457	362.505	212.494	294.593	222.489	332.328	169.099	260.213

+ p<0.10, * p<0.05, ** p<0.01, *** p<0.001

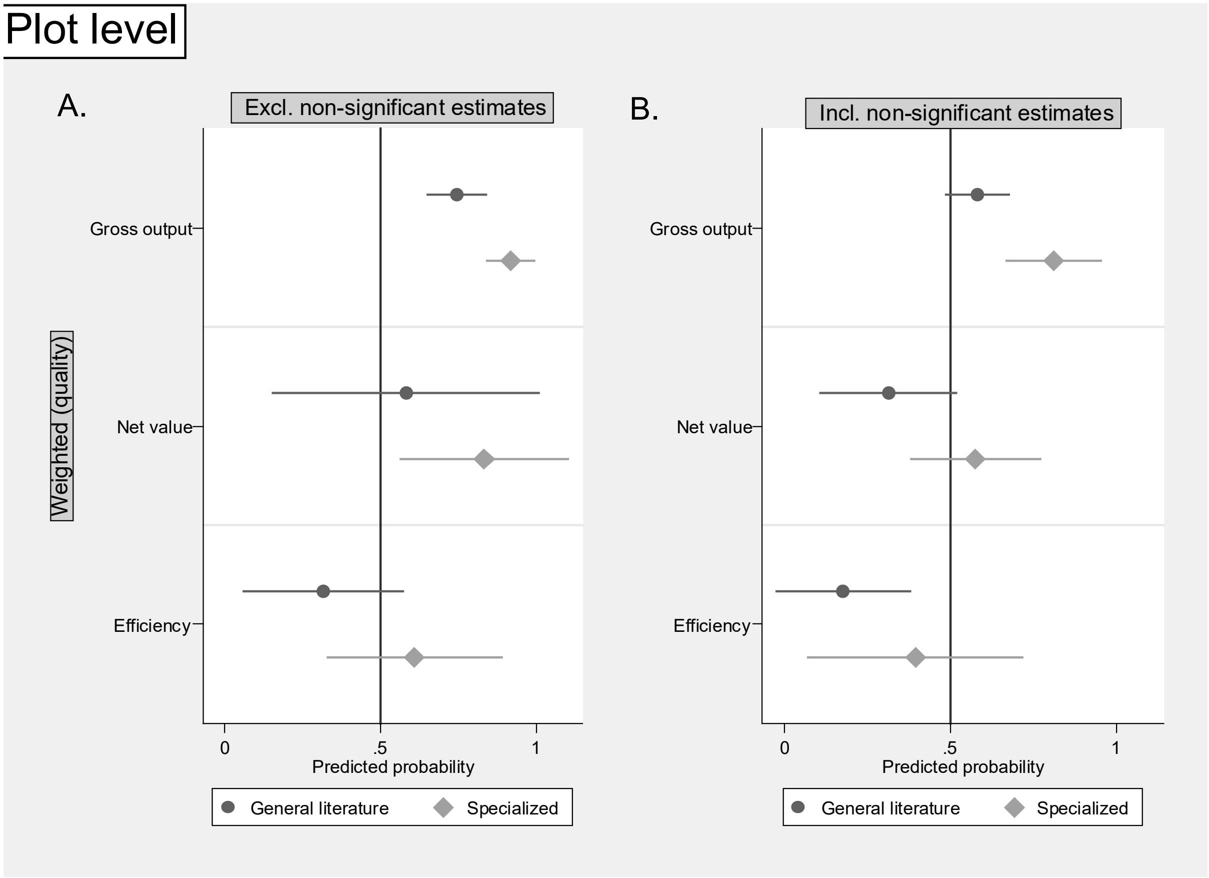

Fig. S 3. Predicted probability of IR, according to whether the study belongs to 'specialized' literature

Plot-level model A excluding non-significant category; Model B including non-significant category. 'Specialized' studies show a higher probability of recording IR than studies from 'general' literature. However, only probabilities using Gross Output significantly differ in both models A and B. Although some difference is perceivable for Net Value and Efficiency, it is not significant as standard errors overlap.

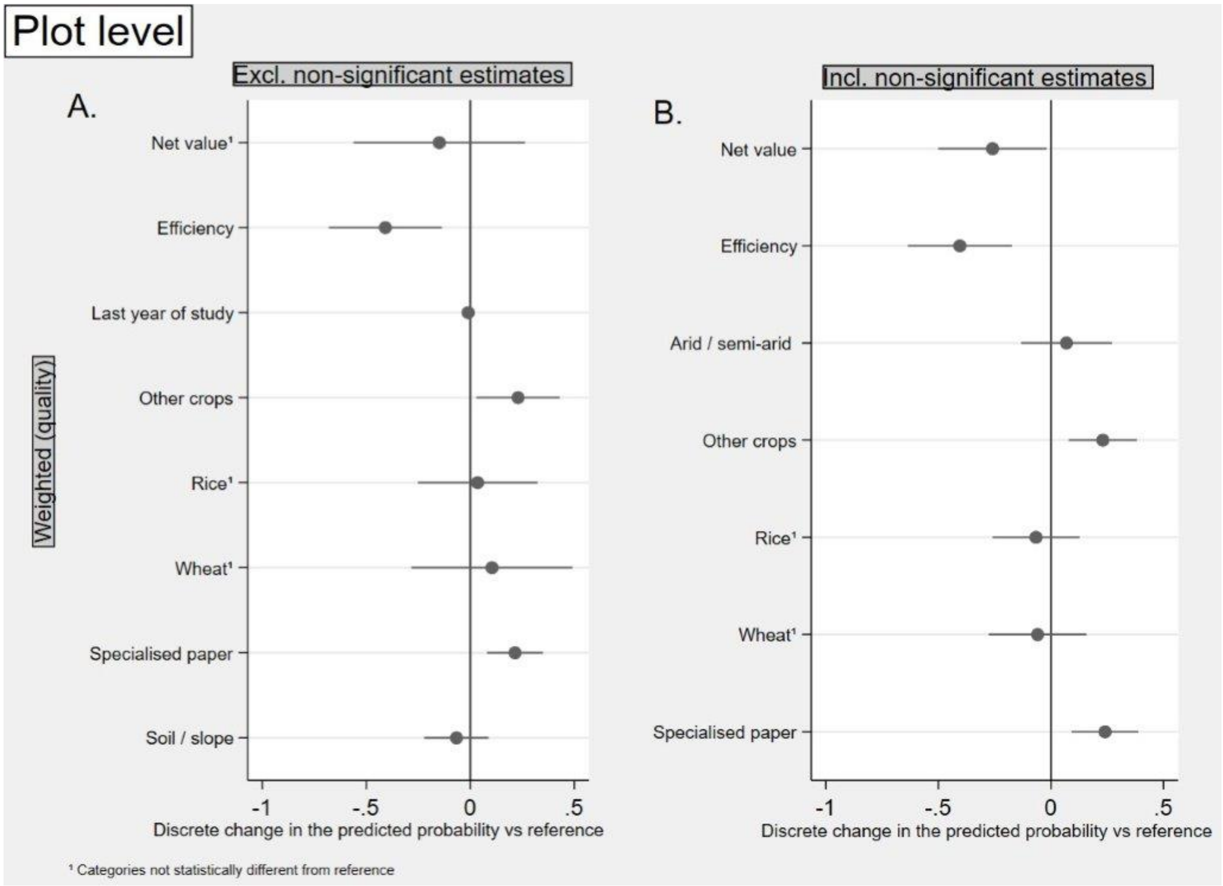

Fig. S 4. Statistically significant discrete change in predicted probabilities of IR, from base level (zero or any unit and category of reference), for significant variables

Plot level model A excluding non-significant category; model B including non-significant category. In all cases, the reference for Net Value and Efficiency is Gross Output; for Other Crops, Rice and Wheat, the reference is Maize. For example, in Model B a study assessing Efficiency is 40 percentage points less likely to record IR than one assessing Gross Output (63%). A study assessing Efficiency in Model B has a 23% probability of recording IR. The remaining variables are dummies (0 or 1). For illustration, Model A suggests that a study accounting for the soil quality or slope of the studied plots is ~10 % less likely to record IR than a study that does not control for this characteristic.

S13: Focusing on the sub-sample of gross output analyses

The significant difference of gross output studies, dominance in the literature and the debates surrounding their misspecification warranted a more detailed exploration.

When only looking at gross output analyses, the overall time trend, the importance of data sources and the tendency of certain crops to favor IR as the orientation are similar to the models estimating all groups of performance indicators presented in the paper. Although significance varies between analyses including non-significant estimates or not. However, two control variables emerge as having some influence on the probability of recording IR, which do not do so in the all sample models. When excluding non-significant estimates (Model A., Table S 7) a study using GPS measurement of land area is more likely to record IR than if only relying on farmers' estimates, in line with the current discussion (*28, 98*). In turn, when contrasting the probability of IR with respect to all other analyzable alternatives (Model B. Table S 7) suggests that IR is 12% less likely to be recorded when a given study controls for the soil quality and/or the slope of the cropped area. This aspect had been raised as important by a series of case studies demonstrating that IR tended to be significantly weakened if not rejected (*97*) but a systematic demonstration of its importance has been elusive when controlling for market imperfections (*22*). Such results suggest that gross output indicator analyses tend to be more sensitive to certain key control variables (i.e. measurement and soil quality control) than alternative performance assessments where such controls do not seem to be playing a major role.

Table S 7. Statistically significant discrete change in the predicted probabilities of IR from base level (zero or any unit and category of reference)

Sub-sample of gross output analyses	Excl. non-significant estimates A.	Incl. non-significant estimates B.
**Data source** (ref: LSMS)		
National Statistics	-0.168*	-0.256***
Ad-hoc surveys	-0.272***	-0.407***
**Warm arid and semi-arid tropics**	-0.025	-0.031
**Time**	-0.017***	-0.020***
**Subsistence farming** (ref: Very small, Mean [0,1] ha)		
Small, Mean (1,2] ha	-0.110	-0.107
Average farm size >2 ha	-0.065	-0.108
**Main crop** (ref: Maize)		
Other crops	0.171*	0.135+
Rice	0.216+	0.106
Wheat	0.098	0.005
**Specialized paper**	-0.058	-0.074
**GPS measurement**	0.198+	0.146
**Inclusion of soil quality and/or slope**	-0.092	-0.126**
**Inclusion of irrigation**	0.009	-0.054
**Inclusion of mechanical and/or animal power**	-0.080	-0.157
**Inclusion of access to credit**	-0.064	-0.077
**N**	346	414

+ p<0.10, * p<0.05, ** p<0.01, *** p<0.001

Farm level, sub-sample limited to gross output analyses. Model A excludes non-significant estimates from the records and Model B includes then to the sample. The list of variables is the same as previous estimation, naturally excluding

the performance indicator group, as this is only focusing on gross output. In all cases, the reference for Net Value and Efficiency is Gross Output; for Other Crops, Rice and Wheat is Maize. Associated logit estimates are presented in the supplementary material.

Farm level gross output only models: Sheet: "*Farm_grossoutput*". Refer to Excel file "Models.xlsx", Sheet: "*Farm_grossoutput*", as Other Supplementary material, for a handier view.

S14: Multicollinearity test

Table S 8. Multicollineality test

Variable	SQRT VIF	VIF	Tolerance	R-square
Gross output	1.52	1.23	0.6561	0.3439
Net value	1.55	1.24	0.6463	0.3537
LSMS surveys	1.57	1.25	0.6381	0.3619
Ad hoc surveys	1.51	1.23	0.6609	0.3391
Warm arid and semi-arid tropics	1.09	1.04	0.9215	0.0785
Time	1.13	1.06	0.8837	0.1163
Land size	1.05	1.03	0.9495	0.0505
Main crop: maize	1.20	1.10	0.8331	0.1669
Main crop: rice	1.20	1.10	0.8325	0.1675
Main crop: wheat	1.14	1.07	0.8755	0.1245
Quartile journals: Q1Q2	1.10	1.05	0.9128	0.0872
Specialized paper	1.26	1.12	0.7946	0.2054
GPS measurement	1.18	1.08	0.8507	0.1493
Inclusion of soil quality and/or slope	1.08	1.04	0.9237	0.0763
Inclusion of irrigation	1.15	1.07	0.8669	0.1331
Inclusion of mechanical and/or animal power	1.03	1.02	0.9697	0.0303
Inclusion of access to credit	1.22	1.10	0.8201	0.1799
**Mean VIF**	**1.23**			

Values of VIF (variance inflation factor) =1 and Tolerance=1 would indicate that the variables are orthogonal to each other and that are completely uncorrelated to each other.

The VIF and the Tolerance (1-R square, resulting from the regression of the other variables over that variable) values do not indicate significant multicollinearity potential among our key variables. VIF (1.23) and Tolerance (~0.64/0.97) are reasonably close to 1.

REFERENCES AND NOTES

1. S. K. Lowder, J. Skoet, T. Raney, The number, size, and distribution of farms, smallholder farms, and family farms worldwide. *World Dev.* **87**, 16–29 (2016).

2. FAO, *The State of Food and Agriculture: Innovation in Family Farming* (FAO, 2014).

3. Alliance for a Green Revolution in Africa, *Africa Agriculture Status Report: Progress Towards Agriculture Transformation in Sub-Saharan Africa* (Alliance for a Green Revolution in Africa, 2016).

4. L. H. Samberg, J. S. Gerber, N. Ramankutty, M. Herrero, P. C. West, Subnational distribution of average farm size and smallholder contributions to global food production. *Environ. Res. Lett.* **11**, 124010 (2016).

5. M. Maertens, B. Minten, J. Swinnen, Modern food supply chains and development: Evidence from horticulture export sectors in Sub-Saharan Africa. *Dev. Policy Rev.* **30**, 473–497 (2012).

6. T. Kuma, M. Dereje, K. Hirvonen, B. Minten, Cash crops and food security: Evidence from Ethiopian smallholder coffee producers. *J. Dev. Stud.* **55**, 1267–1284 (2019).

7. N. E. Rada, K. O. Fuglie, New perspectives on farm size and productivity. *Food Policy* **84**, 147–152 (2019).

8. R. Eastwood, M. Lipton, A. Newell, Farm size, in *Handbook of Agricultural Economics*, R. Evenson, P. Pingali, Eds. (Elsevier, 2010), vol. 4, pp. 3323–3397.

9. H. Binswanger-Mkhize, K. Deininger, G. Feder, Power, distortions, revolt and reform in agricultural land relations, in *Handbook of Development Economics*, J. Behrman, T. Srinivasan, Eds. (Elsevier, 1995), vol. 3, pp. 2659–2772.

10. K. Fuglie, S. L. Wang, Productivity growth in global agriculture shifting to developing countries. *Choices* **27**, 1–7 (2012).

11. T. Adamopoulos, D. Restuccia, The size distribution of farms and international productivity differences. *Am. Econ. Rev.* **104**, 1667–1697 (2014).

12. HLPE, *Investing in Smallholder Agriculture for Food Security. A Report by the High Level Panel of Experts on Food Security and Nutrition* (FAO, 2013).

13. A. V. Chayanov, in *The Theory of Peasant Economy*, D. Thorner, B. Kerblay, R. E. F. Smith, Eds. (American Economic Association, 1966).

14. A. K. Sen, An aspect of Indian agriculture. *Econ. Weekly* **14**, 4–6 (1962).

15. A. K. Sen, Peasants and dualism with or without surplus labor. *J. Political Econ.* **74**, 425–450 (1966).

16. P. K. Bardhan, Size, productivity, and returns to scale: An analysis of farm-level data in Indian agriculture. *J. Political Econ.* **81**, 1370–1386 (1973).

17. M. R. Carter, Identification of the inverse relationship between farm size and productivity: An empirical analysis of peasant agricultural production. *Oxf. Econ. Pap.* **36**, 131–145 (1984).

18. R. Heltberg, Rural market imperfections and the farm size—productivity relationship: Evidence from Pakistan. *World Dev.* **26**, 1807–1826 (1998).

19. A. Hoque, Farm size and economic-allocative efficiency in Bangladesh agriculture. *Appl. Econ.* **20**, 1353–1368 (1988).

20. R. A. Berry, W. R. Cline, *Agrarian Structure and Productivity in Developing Countries: A Study Prepared for the International Labour Office within the Framework of the World Employment Programme* (Johns Hopkins Univ. Press, 1979).

21. W. C. Thiesenhusen, J. Melmed-Sanjak, Brazil's agrarian structure: Changes from 1970 through 1980. *World Dev.* **18**, 393–415 (1990).

22. C. B. Barrett, M. F. Bellemare, J. Y. Hou, Reconsidering conventional explanations of the inverse productivity–size relationship. *World Dev.* **38**, 88–97 (2010).

23. C. Carletto, S. Savastano, A. Zezza, Fact or artifact: The impact of measurement errors on the farm size–productivity relationship. *J. Dev. Econ.* **103**, 254–261 (2013).

24. K. Otsuka, Y. Liu, F. Yamauchi, Growing advantage of large farms in Asia and its implications for global food security. *Glob. Food Secur.* **11**, 5–10 (2016).

25. C. B. Barrett, On price risk and the inverse farm size-productivity relationship. *J. Dev. Econ.* **51**, 193–215 (1996).

26. M. Eswaran, A. Kotwal, Access to capital and agrarian production organisation. *Econ. J.* **96**, 482–498 (1986).

27. A. D. Foster, M. R. Rosenzweig, "Are there too many farms in the world? Labor-market transaction costs, machine capacities and optimal farm size," Working paper no. 23909, National Bureau of Economic Research, October 2017.

28. K. Deininger, S. Jin, Y. Liu, S. K. Singh, Can labor market imperfections explain changes in the inverse farm size-productivity relationship?: Longitudinal evidence from rural India. *Land Econ.* **94**, 239–258 (2018).

29. M. R. Carter, K. D. Wiebe, Access to capital and its impact on agrarian structure and productivity in Kenya. *Am. J. Agric. Econ.* **72**, 1146–1150 (1990).

30. G. Feder, The relation between farm size and farm productivity: The role of family labor, supervision and credit constraints. *J. Dev. Econ.* **18**, 297–313 (1985).

31. M. A. Taslim, Supervision problems and the size-productivity relation in Bangladesh agriculture. *Oxf. Bull. Econ. Stat.* **51**, 55–71 (1989).

32. World Bank, *World Development Report 2008: Agriculture for Development* (World Bank Group, 2007).

33. FAO, *The State of Food and Agriculture: Innovation in Family Farming* (FAO, 2014).

34. D. F. Larson, K. Otsuka, T. Matsumoto, T. Kilic, Should African rural development strategies depend on smallholder farms? An exploration of the inverse-productivity hypothesis. *Agric. Econ.* **45**, 355–367 (2014).

35. D. Gollin, "Farm size and productivity: Lessons from recent literature," *IFAD Research Series* (no. 34) (2019).

36. K. Otsuka, Y. Liu, F. Yamauchi, The future of small farms in Asia. *Dev. Policy Rev.* **34**, 441–461 (2016).

37. Y. Liu, W. J. Violette, C. B. Barrett, "Structural transformation and intertemporal evolution of real wages, machine use, and farm size–productivity relationships in Vietnam," Discussion paper 1525, IFPRI, April 2016.

38. M. Gautam, M. Ahmed, Too small to be beautiful? The farm size and productivity relationship in Bangladesh. *Food Policy* **84**, 165–175 (2019).

39. S. DeSilva, Access to markets and farm efficiency: A study of Bicol rice farms over two decades, in *Technology, Innovations and Economic Development: Essays in Honour of Robert E. Evenson*, L. Singh, K. J. Joseph, D. K. N. Johnson, Eds. (SAGE Publications Ltd., 2015), pp. 147.

40. S. Savastano, P. Scandizzo, Optimal farm size in an uncertain land market: The case of Kyrgyz Republic. *Agric. Econ.* **40**, 745–758 (2009).

41. J. Wang, K. Z. Chen, S. D. Gupta, Z. Huang, Is small still beautiful? A comparative study of rice farm size and productivity in China and India. *China Agric. Econ. Rev.* **7**, 484–509 (2015).

42. P. L. Scandizzo, S. Savastano, Revisiting the farm size–productivity relationship: New evidence from Sub-Saharan African countries, in *Agriculture and Rural Development in a*

Globalizing World: Challenges and Opportunities, P. Pingali, G. Feder, Eds. (Routledge, 2017), chap. 3, pp. 26–55.

43. P. Collier, S. Dercon, African agriculture in 50 years: Smallholders in a rapidly changing world? *World Dev.* **63**, 92–101 (2014).

44. J. J. Assunção, L. H. B. Braido, Testing household-specific explanations for the inverse productivity relationship. *Am. J. Agric. Econ.* **89**, 980–990 (2007).

45. J. J. Assunção, M. Ghatak, Can unobserved heterogeneity in farmer ability explain the inverse relationship between farm size and productivity. *Econ. Lett.* **80**, 189–194 (2003).

46. D. Benjamin, Can unobserved land quality explain the inverse productivity relationship? *J. Dev. Econ.* **46**, 51–84 (1995).

47. S. S. Bhalla, P. Roy, Mis-specification in farm productivity analysis: The role of land quality. *Oxf. Econ. Pap.* **40**, 55–73 (1988).

48. D. Gollin, C. Udry, "Heterogeneity, measurement error and misallocation: Evidence from African agriculture," Working paper no. 25440, National Bureau of Economic Research, January 2019.

49. S. Desiere, D. Jolliffe, Land productivity and plot size: Is measurement error driving the inverse relationship? *J. Dev. Econ.* **130**, 84–98 (2018).

50. S. Gourlay, T. Kilic, D. Lobell, paper presented at the Center for the Study of African Economies (CSAE) Conference, Oxford, UK, 20 March 2017.

51. C. Bizimana, W. L. Nieuwoudt, S. R. D. Ferrer, Farm size, land fragmentation and economic efficiency in Southern Rwanda. *Agrekon* **43**, 244–262 (2004).

52. T. S. Jayne, M. Muyanga, A. Wineman, H. Ghebru, C. Stevens, M. Stickler, A. Chapoto, W. Anseeuw, D. van der Westhuizen, D. Nyange, Are medium-scale farms driving agricultural transformation in Sub-Saharan Africa? *Agric. Econ.* **50**, 75–95 (2019).

53. M. Muyanga, T. S. Jayne, Revisiting the farm size-productivity relationship based on a relatively wide range of farm sizes: Evidence from Kenya. *Am. J. Agric. Econ.* **101**, 1140–1163 (2019).

54. T. S. Jayne, J. Chamberlin, L. Traub, N. Sitko, M. Muyanga, F. K. Yeboah, W. Anseeuw, A. Chapoto, A. Wineman, C. Nkonde, R. Kachule, Africa's changing farm size distribution patterns: The rise of medium-scale farms. *Agric. Econ.* **47**, 197–214 (2016).

55. D. F. Larson, R. Muraoka, K. Otsuka, Why African rural development strategies must depend on small farms. *Glob. Food Secur.* **10**, 39–51 (2016).

56. D. Moher, L. Shamseer, M. Clarke, D. Ghersi, A. Liberati, M. Petticrew, P. Shekelle, L. A. Stewart; PRISMA-P Group, Preferred reporting items for systematic review and meta-analysis protocols (PRISMA-P) 2015 statement. *Syst. Rev.* **4**, 1 (2015).

57. D. Moher, A. Liberati, J. Tetzlaff, D. G. Altman; PRISMA Group, Preferred reporting items for systematic reviews and meta-analyses: The PRISMA statement. *PLOS Med.* **6**, e1000097 (2009).

58. T. D. Stanley, H. Doucouliagos, M. Giles, J. H. Heckemeyer, R. J. Johnston, P. Laroche, J. P. Nelson, M. Paldam, J. Poot, G. Pugh, R. S. Rosenberger, K. Rost, Meta-analysis of economics research reporting guidelines. *J. Econ. Surv.* **27**, 390–394 (2013).

59. Y. Sheng, J. Ding, J. Huang, The relationship between farm size and productivity in agriculture: Evidence from maize production in Northern China. *Am. J. Agric. Econ.* **101**, 790–806 (2019).

60. W. Vellema, A. Buritica Casanova, C. Gonzalez, M. D'Haese, The effect of specialty coffee certification on household livelihood strategies and specialisation. *Food Policy* **57**, 13–25 (2015).

61. M. Sheahan, R. Black, T. S. Jayne, Are Kenyan farmers under-utilizing fertilizer? Implications for input intensification strategies and research. *Food Policy* **41**, 39–52 (2013).

62. T. Kilic, A. Palacios-López, M. Goldstein, Caught in a productivity trap: A distributional perspective on gender differences in Malawian agriculture. *World Dev.* **70**, 416–463 (2015).

63. J. Michler, G. Shively, Land tenure, tenure security and farm efficiency: Panel evidence from the Philippines. *J. Agric. Econ.* **66**, 155–169 (2015).

64. M. Closset, B. B. B. Dhehibi, A. Aw-Hassan, Measuring the economic impact of climate change on agriculture: A Ricardian analysis of farmlands in Tajikistan. *Clim. Dev.* **7**, 454–468 (2015).

65. A. Kimhi, Plot size and maize productivity in Zambia: Is there an inverse relationship? *Agric. Econ.* **35**, 1–9 (2006).

66. T. D. Stanley, Wheat from chaff: Meta-analysis as quantitative literature review. *J. Econ. Perspect.* **15**, 131–150 (2001).

67. T. D. Stanley, Meta-regression methods for detecting and estimating empirical effects in the presence of publication selection. *Oxf. Bull. Econ. Stat.* **70**, 103–127 (2008).

68. T. D. Stanley, Beyond publication bias. *J. Econ. Surv.* **19**, 309–345 (2005).

69. C. Nkonde, T. S. Jayne, R. Richardson, F. Place, in *World Bank Land and Poverty Conference 2015*, Washington, DC, 23 to 27 March 2015.

70. R. E. Evenson, D. Gollin, Assessing the impact of the green revolution, 1960 to 2000. *Science* **300**, 758–762 (2003).

71. H. Binswanger-Mkhize, A. F. McCalla, The changing context and prospects for agricultural and rural development in Africa, in *Handbook of Agricultural Economics* (Elsevier, 2010), vol. 4, pp. 3571–3712.

72. P. L. Pingali, Green Revolution: Impacts, limits, and the path ahead. *Proc. Natl. Acad. Sci. U.S.A.* **109**, 12302–12308 (2012).

73. M. Smale, D. Byerlee, T. Jayne, Maize revolutions in Sub-Saharan Africa, in *An African Green Revolution: Finding Ways to Boost Productivity on Small Farms*, K. Otsuka, D. F. Larson, Eds. (Springer, 2013), pp. 165–195.

74. D. F. Larson, R. Muraoka, K. Otsuka, Rural development strategies and Africa's small farms, in *The Role of Smallholder Farms in Food and Nutrition Security*, S. Gomez y Paloma, L. Riesgo, K. Louhichi, Eds. (Springer International Publishing, Cham, 2020), pp. 45–77.

75. K. Takahashi, K. Otsuka, The increasing importance of nonfarm income and the changing use of labor and capital in rice farming: The case of Central Luzon, 1979–2003. *Agric. Econ.* **40**, 231–242 (2009).

76. K. Otsuka, Y. Liu, F. Yamauchi, Factor endowments, wage growth, and changing food self-sufficiency: Evidence from country-level panel data. *Am. J. Agric. Econ.* **95**, 1252–1258 (2013).

77. F. Yamauchi, Rising real wages, mechanization and growing advantage of large farms: Evidence from Indonesia. *Food Policy* **58**, 62–69 (2016).

78. Y. Liu, C. B. Barrett, T. Pham, W. Violette, The intertemporal evolution of agriculture and labor over a rapid structural transformation: Lessons from Vietnam. *Food Policy* **94**, 101913 (2020).

79. X. Wang, F. Yamauchi, K. Otsuka, J. Huang, Wage growth, landholding, and mechanization in Chinese agriculture. *World Dev.* **86**, 30–45 (2016).

80. H. P. Binswanger-Mkhize, S. Savastano, Agricultural intensification: The status in six African countries. *Food Policy* **67**, 26–40 (2017).

81. C. B. Barrett, L. Christiaensen, M. Sheahan, A. Shimeles, On the structural transformation of rural Africa. *J. Afr. Econ.* **26**, i11–i35 (2017).

82. B. Davis, S. Di Giuseppe, A. Zezza, Are African households (not) leaving agriculture? Patterns of households' income sources in rural Sub-Saharan Africa. *Food Policy* **67**, 153–174 (2017).

83. G. Djurfeldt, E. Aryeetey, A. C. Isinika, *African Smallholders: Food Crops, Markets and Policy* (CABI Publishing, 2011).

84. T. S. Jayne, J. Chamberlin, D. D. Headey, Land pressures, the evolution of farming systems, and development strategies in Africa: A synthesis. *Food Policy* **48**, 1–17 (2014).

85. M. Sheahan, C. B. Barrett, Ten striking facts about agricultural input use in Sub-Saharan Africa. *Food Policy* **67**, 12–25 (2017).

86. C. B. Barrett, S. M. Sherlund, A. A. Adesina, Shadow wages, allocative inefficiency, and labor supply in smallholder agriculture. *Agric. Econ.* **38**, 21–34 (2008).

87. N. Sitko, J. Chamberlin, The anatomy of medium-scale farm growth in Zambia: What are the implications for the future of smallholder agriculture? *Land* **4**, 869–887 (2015).

88. S. T. Holden, Policies for improved food security: The roles of land tenure policies and land markets, in *The Role of Smallholder Farms in Food and Nutrition Security*, S. Gomez y Paloma, L. Riesgo, K. Louhichi, Eds. (Springer International Publishing, 2020), pp. 153–169.

89. L. Riesgo, K. Louhichi, S. Gomez y Paloma, Conclusions, in *The Role of Smallholder Farms in Food and Nutrition Security*, S. Gomez y Paloma, L. Riesgo, K. Louhichi, Eds. (Springer International Publishing, Cham, 2020), pp. 247–251.

90. A. de Janvry, E. Sadoulet, Agriculture for development in Africa: Business-as-usual or new departures? *J. Afr. Econ.* **19**, ii7–ii39 (2010).

91. P. Dorosh, J. Thurlow, Beyond agriculture versus non-agriculture: Decomposing sectoral growth–poverty linkages in five African countries. *World Dev.* **109**, 440–451 (2018).

92. S. Dercon, D. Gollin, Agriculture in African development: Theories and strategies. *Annu. Rev. Resour. Econ.* **6**, 471–492 (2014).

93. R. Frelat, S. Lopez-Ridaura, K. E. Giller, M. Herrero, S. Douxchamps, A. A. Djurfeldt, O. Erenstein, B. Henderson, M. Kassie, B. K. Paul, C. Rigolot, R. S. Ritzema, D. Rodriguez, P. J. A. van Asten, M. T. van Wijk, Drivers of household food availability in Sub-Saharan Africa based on big data from small farms. *Proc. Natl. Acad. Sci. U.S.A.* **113**, 458–463 (2016).

94. K. Otsuka, T. Yamano, *The Role of Rural Labor Markets in Poverty Reduction: Evidence from Asia and East Africa* (World Bank, 2008).

95. B. O. Muthén, A. Satorra, Complex sample data in structural equation modeling. *Sociol. Methodol.* **25**, 267–316 (1995).

96. N. Sommet, D. Morselli, Keep calm and lean multilevel logistic modeling: A simplified three-step procedure using Stata, R, Mplus, and SPSS. *Int. Rev. Soc. Psychol.* **30**, 203–218 (2017).

97. T. Kilic, A. Zezza, C. Carletto, S. Savastano, Missing(ness) in action: Selectivity bias in GPS-based land area measurements. *World Dev.* **92**, 143–157 (2017).

98. R. L. Lamb, Inverse productivity: Land quality, labor markets, and measurement error. *J. Dev. Econ.* **71**, 71–95 (2003).
